# Where east meets west: Phylogeography of the high Arctic North American brant goose

**DOI:** 10.1002/ece3.11245

**Published:** 2024-04-10

**Authors:** Robert E. Wilson, W. Sean Boyd, Sarah A. Sonsthagen, David H. Ward, Preben Clausen, Kathryn M. Dickson, Barwolt S. Ebbinge, Gudmundur A. Gudmundsson, George K. Sage, Jolene R. Rearick, Dirk V. Derksen, Sandra L. Talbot

**Affiliations:** ^1^ School of Natural Resources University of Nebraska‐Lincoln Lincoln Nebraska USA; ^2^ Nebraska State Museum University of Nebraska‐Lincoln Lincoln Nebraska USA; ^3^ Environment and Climate Change Canada Science and Technology Branch Delta British Columbia Canada; ^4^ U.S. Geological Survey, Nebraska Cooperative Fish and Wildlife Research Unit, School of Natural Resources University of Nebraska‐Lincoln Lincoln Nebraska USA; ^5^ U.S. Geological Survey Alaska Science Center Anchorage Alaska USA; ^6^ Department of Ecoscience Aarhus University Aarhus Denmark; ^7^ Canadian Wildlife Service Environment and Climate Change Canada Ottawa Ontario Canada; ^8^ Animal Ecology Alterra Wageningen‐UR Wageningen The Netherlands; ^9^ Icelandic Institute of Natural History Gardabaer Iceland; ^10^ Far Northwestern Institute of Art and Science Anchorage Alaska USA; ^11^ Independent Researcher Little Rock Arkansas USA; ^12^ Alaska Center for Conservation Science University of Alaska Anchorage Alaska USA

**Keywords:** brant geese, *Branta bernicla*, contact zones, genetic structure, refugia

## Abstract

Genetic variation in Arctic species is often influenced by vicariance during the Pleistocene, as ice sheets fragmented the landscape and displaced populations to low‐ and high‐latitude refugia. The formation of secondary contact or suture zones during periods of ice sheet retraction has important consequences on genetic diversity by facilitating genetic connectivity between formerly isolated populations. Brant geese (*Branta bernicla*) are a maritime migratory waterfowl (Anseriformes) species that almost exclusively uses coastal habitats. Within North America, brant geese are characterized by two phenotypically distinct subspecies that utilize disjunct breeding and wintering areas in the northern Pacific and Atlantic. In the Western High Arctic of Canada, brant geese consist of individuals with an intermediate phenotype that are rarely observed nesting outside this region. We examined the genetic structure of brant geese populations from each subspecies and areas consisting of intermediate phenotypes using mitochondrial DNA (mtDNA) control region sequence data and microsatellite loci. We found a strong east–west partition in both marker types consistent with refugial populations. Within subspecies, structure was also observed at mtDNA while microsatellite data suggested the presence of only two distinct genetic clusters. The Western High Arctic (WHA) appears to be a secondary contact zone for both Atlantic and Pacific lineages as mtDNA and nuclear genotypes were assigned to both subspecies, and admixed individuals were observed in this region. The mtDNA sequence data outside WHA suggests no or very restricted intermixing between Atlantic and Pacific wintering populations which is consistent with published banding and telemetry data. Our study indicates that, although brant geese in the WHA are not a genetically distinct lineage, this region may act as a reservoir of genetic diversity and may be an area of high conservation value given the potential of low reproductive output in this species.

## Graphical abstract

Photographs of the three different plumage coloration types of brant within North America: Pacific Black (left), Western High Arctic (middle), and Atlantic (right). Photograph credit: Maynard Axelson.

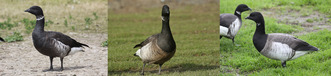

## 1 INTRODUCTION

The distribution and patterns of genetic diversity of northern latitude species were greatly influenced by fluctuating climatic conditions associated with Pleistocene glacial cycles (Hewitt, 2001, 2004). Over 20 glacial cycles have been recorded (Williams et al., 1998), resulting in major range shifts and fluctuations in population demography of Arctic species whose ecological and physiological characteristics determined the magnitude of impact (Stewart et al., 2010). As species distributions shifted in association with the expansion and retreat of ice sheets, landscape barriers would have isolated populations allowing evolutionary processes (e.g., genetic drift and local adaptation) to act independently on each population (Cumer et al., 2022). After ice retreat and a period of evolutionary divergence in isolation (i.e., refugial confinement), formerly allopatric populations may reconnect at a secondary contact location (Taberlet et al., 1998). Over time, through periods of cyclical isolation and secondary contact, narrow hybrid zones may form between populations and could result in the complete fusion of lineages (Bertl et al., 2018; Maier et al., 2019). Thus, the formation of these secondary contact locations and connectivity to “parental” populations have important consequences for historical and contemporary distributions of genetic diversity.

Geese that nest in the Arctic are considered keystone herbivores whose population trends and distribution are influenced by environmental conditions (Cooch et al., 2001). Although many species experienced significant population growth beginning in the 1970s, North American brant geese (hereafter referred to as brant; *Branta bernicla*) did not undergo a marked sustained increase in population size but rather generally declined with variable fluctuations between the 1960s and 1990s (Lewis et al., 2020; Sedinger et al., 2019). Brant productivity is highly variable across years, especially in the Arctic (Ward et al., 2018), with pairs forgoing breeding in years when environmental conditions are unfavorable (Barry, 1962; Ebbinge & Spaans, 1995). In the subarctic, such as the Yukon‐Kuskokwim Delta (YKD), Alaska, low nesting success is largely driven by high fox (*Vulpes* spp.) predation, and (or) extreme flood and El Niño events (see Sedinger et al., 2006). Outside of the nesting grounds, North American brant vital rates are influenced by hunting and the abundance of eelgrass (*Zostera marina* L.) on staging and wintering sites as they rely heavily on this single food during the nonbreeding season (Ganter, 2000; Ward et al., 2005). This contrasts with nearly all other geese that exploit agricultural crops as a high energy food source. Due to recent declines and distinct factors that influence population demography in brant, understanding how populations are structured and the potential genetic connectivity among areas is important for taxonomic decision‐making and development of conservation and management strategies.

**FIGURE 1:**
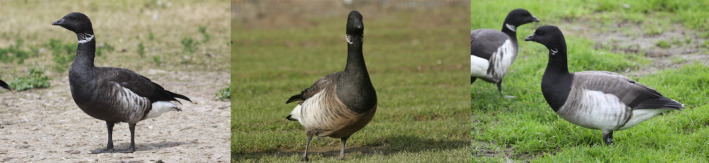
Photographs of the three different plumage coloration types of brant within North America: Pacific Black (*nigricans*, left), Western High Arctic (*nigricans*, middle), and Atlantic (*hrota*, right). Photograph credit: Maynard Axelson.

Brant are a maritime goose species comprised of three distinct subspecies (*B. b. bernicla*, *B. b. nigricans*, and *B. b. hrota*) based on discreteness of nesting and wintering area locations and plumage, especially the color of the belly and the completeness of the white necklace (see Lewis et al., 2020 for subspecific descriptions; Figure 1). In Europe, the dark‐bellied brant (*B. b. bernicla*; referred to as *bernicla* hereafter) are the most abundant subspecies, nesting in western Siberia primarily on the Taimyr Peninsula (Ebbinge et al., 1999; Lewis et al., 2020). This subspecies winters in western Europe including Denmark, The Netherlands, England, and France. Within North America, there is a strong east–west migratory divide separating two subspecies, the light‐bellied brant (*B. b. hrota*; referred to as *hrota* hereafter) and black brant (*B. b. nigricans*; referred to as *nigricans* hereafter). Spatial segregation for most of the annual cycle (e.g., O'Briain et al., 1998) has led to the identification of “ecological stocks” that have influenced management decisions, including harvest, within and across subspecies (Castelli et al., 2011; Pacific Flyway Council, 2018). The light‐bellied brant in eastern North America has been divided into populations nesting in the low to mid‐Arctic Canada that winter along the western Atlantic coast (“Atlantic brant”) and geese nesting within the “Eastern High Arctic” (EHA; e.g., Bathurst Island) Canada that overwinter primarily in Ireland. Small populations of *hrota* also breed in Svalbard, Franz Josef Land and northeastern Greenland and winter in western Europe mainly in Denmark and England (Clausen et al., 1999; Lewis et al., 2020). In western North America, *nigricans* are divided into “Pacific black brant” that nest primarily in subarctic and low and high arctic Russia, Alaska, and Canada, and winter from Alaska to Mexico, and “western high Arctic” (WHA) brant nesting on the Parry Islands, Canada, and overwinter in the Salish Sea, primarily in Washington, United States of America (Boyd et al., 2013; Boyd & Maltby, 1979; Reed, Davison, et al., 1989). Although the three subspecies and North America “ecological” stocks in general have areas of their distributions where one subspecies predominates, there are areas of overlap, particularly between *bernicla* and *hrota* and *nigricans* (Baldassare, 2014). For example, *bernicla* is known to form mixed nesting colonies with *nigricans* in the western Lena River and Olenyok River Deltas of central Siberia (Hellquist et al., 2018; Syroechkovski et al., 1998) and overlap with *hrota* at wintering areas in western Europe (e.g., France, The Netherlands, and England; Dalloyau, 2022; Koffijberg et al., 2013). The three subspecies may also co‐occur as mixed pairs in France, though rare for *nigricans* (Dalloyau, 2022), suggesting subspecies are not reproductively isolated.

The WHA brant are often referred to as “gray‐bellied” brant due to their intermediate belly color between light‐bellied (*hrota*) and black brant (*nigricans*) and are one of the smallest Arctic goose populations (~8000–11,000 individuals; Fox & Leafloor, 2018; Lewis et al., 2020). Gray‐bellied brant are primarily restricted to the Parry Islands region where it makes up approximately 74% of the population and are rarely observed nesting in other areas (Boyd & Maltby, 1979; Reed, Davison, et al., 1989). The general lack of nesting records outside the Parry Islands region suggests this intermediate plumage is maintained either through assortative mating and (or) continued interbreeding with either or both subspecies to form a narrow stable hybrid zone. Previous genetic research using a single genetic marker (mitochondrial [mtDNA] restriction fragment length polymorphism data; Shields, 1990) suggested that the gray‐bellied brant may be an isolated population that diverged around 400,000 years ago. However, this is based on a small sample size (19 total with only 4 from the WHA) so the mechanisms maintaining this intermediate body plumage coloration in this confined region are still unknown.

Here we provide the first range‐wide assessment within North America of how genetic diversity is distributed across the Arctic among and within brant populations using genotypic data from 12 microsatellite loci and sequence data from the mitochondrial DNA (mtDNA) control region. Given the strong Pacific‐Atlantic migratory divide and an estimated divergence date of around 1 million years ago (Ottenburghs et al., 2016), we hypothesize that each subspecies will be distinct at both marker types. If WHA brant were isolated for an extended period (e.g., little or no gene flow with other areas), as suggested by Shields (1990), we would expect that samples from this area may have had sufficient time to form a distinctive genetic group. Alternatively, if WHA brant are a result of admixture between Pacific black and Atlantic or EHA brant lineages, either through historical secondary contact and (or) contemporary hybridization, then we would expect samples from this region to show a range of genetic similarities to both subspecies in the nuclear genome (microsatellites) and mtDNA haplotypes shared with both subspecies. Furthermore, failed and nonbreeding brant, in particular black brant, undertake long molt migrations where molting concentrations contain individuals from multiple nesting colonies (Bollinger & Derksen, 1996; Portenko, 1981; Ward et al., 1993). Molting sites have been proposed as important locales for the exchange of individuals across flyways in other geese such as the greater white‐fronted goose (*Anser albifrons*; Kölzsch et al., 2019) that likely aids in maintaining genetic connectivity among disparate nesting areas (Wilson et al., 2022). As brant pair bonds are likely formed away from nesting areas, admixture of different populations and subspecies, as well as any intra‐individual variance in migration routes, may provide opportunities for dispersal and ultimately lead to a lack of genetic structure across broad regions as observed in many duck species (e.g., Brown et al., 2020; Sonsthagen et al., 2019). Therefore, if there is an asymmetrical genetic signature of backcrossing to one or the other subspecies or ecological stocks, we expect it will be more prevalent in the Pacific black brant than in the eastern Canadian populations given increased opportunities to form inter‐stock pair bonds due to sympatry on molting, staging, and wintering grounds. Lastly, to explore potential admixture with *bernicla* outside of North America, we included breeding samples from Lena River in central Siberia and wintering samples from The Netherlands where both *nigricans* and *hrota* are known to intermix with the third subspecies *bernicla*.

## 2 METHODS

### 2.1 Samples

**FIGURE 2:**
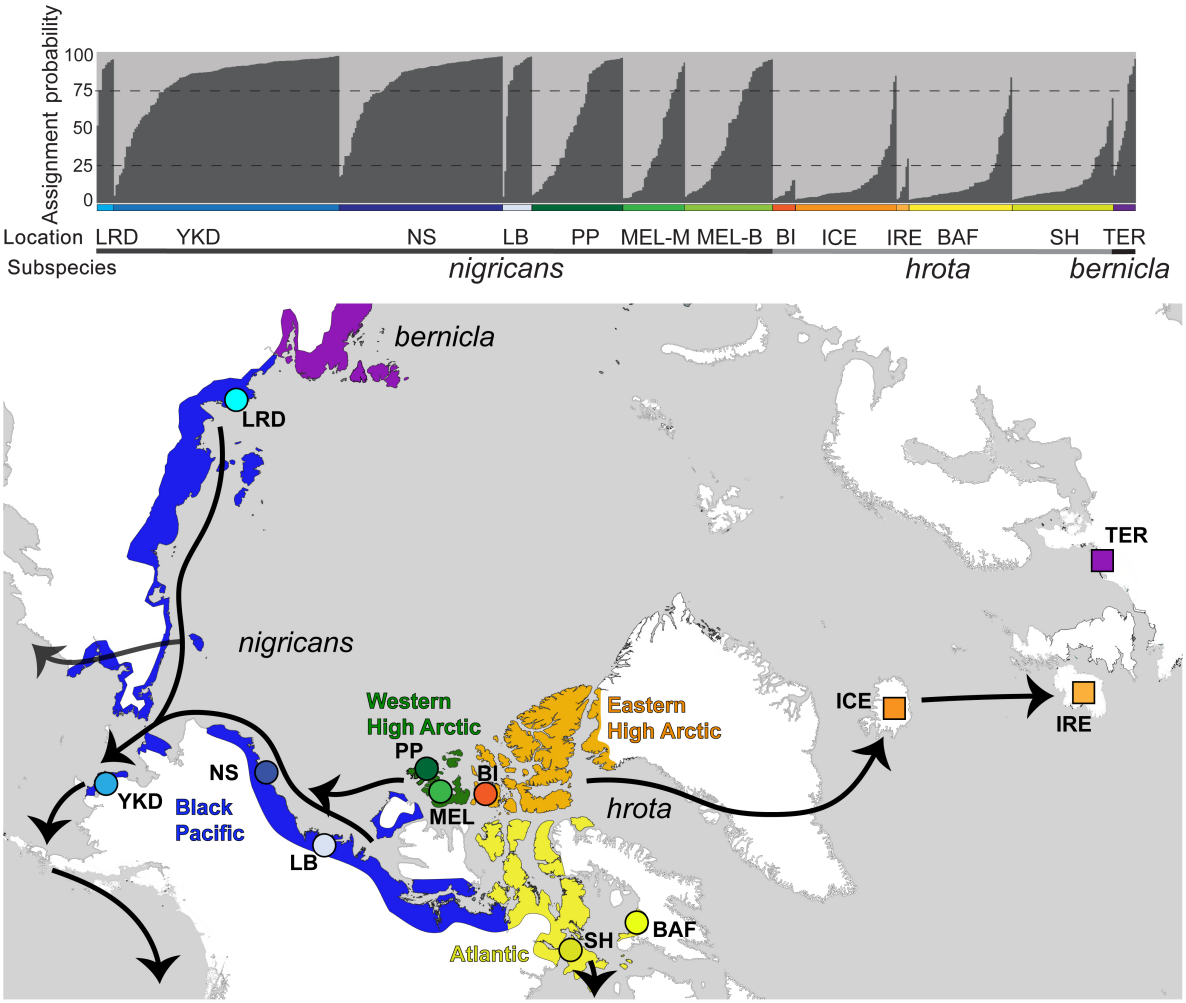
Population subdivision of brant geese (*Brant bernicla*) based on STRUCTURE (top panel) with breeding distribution (color shading) and subspecies/ecological stocks designations indicated by different color shading (bottom panel). STRUCTURE plot shows average assignment probability based on microsatellite data for *K* = 2. See Table S3 for proportion of samples that have *nigricans*‐like genotypes (>75% assignment to dark gray cluster), *hrota*‐like (>75% to light gray cluster), and to uncertain (Admixed group, less than 75% assignment to either group). Color blocks in the top panel correspond to color circles (molting or nesting sample locations) or squares (staging or wintering sample locations) in the bottom panel. Location of brant breeding populations representing the three subspecies (*nigricans*, *hrota*, and *bernicla*) and four ecological stocks (Pacific Black [blue; YKD, NS, LB, LRD], Western High Arctic [green; PP, MEL], Eastern High Arctic [orange: BI, ICE, IRE], and Atlantic [yellow; BAF, SH]). Arrows indicate general migration pathways to staging and wintering areas. Collection location abbreviations are defined in Section 2 and Table 1.

Blood, feather, eggshell membrane, or frozen muscle were opportunistically obtained from 567 brant representing the three subspecies and the Western High Arctic stock (Figure 2; Table 1): *nigricans* (Yukon‐Kuskokwim Delta [YKD] and the North Slope [NS] of Alaska, Liverpool Bay [LB], Northwest Territories, Canada, and Sagastyr Island in the central Lena River Delta [LRD], central Arctic coast of Russia); *hrota* (Baffin [BAF], Southampton [SH], and Bathurst [BI] islands, Canada, wintering birds collected in Ireland [IRE], and spring migrants collected in Iceland [ICE – known to breed in Eastern High Arctic Canada (e.g., Bathurst Island) and northwest Greenland; Lewis et al., 2020]); wintering *bernicla* (Terschelling Island [TER], The Netherlands); and WHA brant (Prince Patrick [PP] and Melville [MEL] islands). Within the YKD, samples were taken from four of the five primary colonies: Kokechik Bay (KOK), Tutakoke River (TR), Kigigak Island (KIG), and Baird Inlet (BAI). Sampling on the NS occurred at the Colville River Delta (COL), the largest colony in the Arctic, and at more dispersed colonies near Ugnuravik (UG) and Oliktok (OL) rivers, Teshekpuk Lake (TESH), Utqiagvik (UT), and Prudhoe Bay (PB). Samples were collected between 1991–1992 (PB, COL), and 2000–2006 (all other locations) and were derived from breeding individuals (BAF, SH, COL, TR, BAI, KOK, KIG, LB, and LRD), wintering birds (IRE, TER), spring staging migrants (ICE), or individuals comprising molting aggregates with families (brood drives: MEL, BI) or without families (adults only: OL, UG, UT, PB, TESH, MEL, PP). Specimen locality details are available in Sonsthagen et al. (2024).

**TABLE 1:** Genetic diversity and fluctuations in population demography at the mtDNA control region and 12 autosomal microsatellite loci among 13 populations/aggregations of brant in North America.

Population/ecological stock	Map	Mitochondrial DNA							Microsatellites							
		*N*	*H*	PH	*h* (SD)	*π* (SD)	*F*_S_	*D*	*N*	*A*	AR	PA	*H*_O_ (SD)	*H*_E_ (SD)	*F*_IS_	DC^a^
*B. bernicla hrota*																
Atlantic Brant																
Baffin Island, Canada	BAF	54	23	10	0.917 (0.025)	0.0118 (0.0068)	**−11.7**	−1.2	60	4.9	2.7	3	0.332 (0.018)	0.350 (0.088)	0.051	**Hdef**
Southampton Island, Canada	SH	30	18	4	0.963 (0.016)	0.0181 (0.0101)	**−5.9**	0.1	59	4.8	2.7	0	0.345 (0.018)	0.348 (0.085)	0.009	**Hdef**
Eastern High Arctic Brant																
Ireland (wintering)	IRE	7	4	3	0.809 (0.130)	0.0095 (0.0065)	0.5	−0.2	7	2.9	2.8	0	0.401 (0.054)	0.377 (0.083)	−0.071	Eq
Iceland (spring migrants)	ICE	25	19	14	0.970 (0.022)	0.0222 (0.0121)	**−8.1**	0.2	59	4.9	2.8	2	0.365 (0.018)	0.369 (0.089)	0.010	**Hdef**
Bathurst Island, Canada	BI	12	8	6	0.924 (0.058)	0.0149 (0.0089)	−1.5	−0.3	13	3.3	2.7	0	0.372 (0.039)	0.373 (0.097)	0.002	Eq
*B. bernicla nigricans*																
Pacific Black Brant																
Liverpool Bay, Canada	LB	‐	‐	‐	‐	‐	‐	‐	17	4.3	3.1	0	0.411 (0.035)	0.429 (0.082)	0.042	**Hdef**
North Slope, Alaska^b^	NS	65	26	13	0.886 (0.031)	0.0247 (0.0130)	−5.5	0.9	95	6.6	3.1	1	0.407 (0.015)	0.417 (0.085)	0.024	**Hdef**
Yukon‐Kuskokwim Delta, Alaska^c^	YKD	121	31	23	0.644 (0.052)	0.0102 (0.0059)	**−18.8**	−0.8	131	5.4	3.2	2	0.422 (0.013)	0.432 (0.084)	0.024	**Hdef**
Lena River Delta, Russia	LRD	9	8	7	0.972 (0.064)	0.0270 (0.0157)	−1.6	0.5	10	6.9	3.1	0	0.422 (0.013)	0.432 (0.084)	−0.042	Eq
Western High Arctic Brant																
Melville Island, Canada (breeders)	MEL‐B	16	15	1	0.992 (0.025)	0.0394 (0.0211)	**−5.9**	0.9	36	4.5	2.9	0	0.350 (0.023)	0.380 (0.092)	0.081	Eq
Melville Island, Canada (molting)	MEL‐M	6	5	3	0.933 (0.122)	0.0237 (0.0150)	0.2	0.2	51	4.8	2.9	0	0.378 (0.023)	0.387 (0.090)	0.025	Eq
Prince Patrick Island, Canada (molting)	PP	32	18	8	0.929 (0.028)	0.0351 (0.0182)	−1.9	1.3	53	5.9	3.0	1	0.385 (0.019)	0.391 (0.088)	0.016	**Hdef**
*B. bernicla bernicla*																
Terschelling, The Netherlands	TER	9	7	7	0.917 (0.092)	0.0122 (0.0077)	−2.0	−0.2	13	4.1	3.1	0	0.404 (0.039)	0.399 (0.093)	−0.011	Eq

*Note*: Comparative data from brant of Europe (Terschelling) and eastern Russia (LRD) are shown. Mitochondrial DNA: *H* = number of haplotypes observed; PH = number of private haplotypes; *h* = haplotype diversity; *π* = nucleotide diversity; *F*_S_ = Fu's *F*_S_; *D* = Tajima's *D*. Microsatellites: *A* = average number of alleles; AR = allelic richness: PA = number of private alleles; *H*_O_ = observed heterozygosity; *H*_E_ = expected heterozygosity; *F*_IS_ = inbreeding coefficient and variance in parentheses; DC = demographic change analysis under the stepwise mutation model. Values that are significantly different from zero are in bold text. Map refers to population abbreviations in Figure 1.

^a^ Significant heterozygote deficiency (Hdef) indicates population growth; non‐significant population estimates indicate population equilibrium (Eq).

^b^ Includes sampling from Alaska, USA: Colville River Delta, Ugnuravik and Oliktok rivers, Teshekpuk Lake, Utqiagvik, and Prudhoe Bay.

^c^ Includes sampling from Alaska USA: Kokechik Bay, Tutakoke River, Kigigak Island and Baird Inlet.

### 2.2 Laboratory techniques

DNA extractions followed Handel et al. (2006) for blood and eggshell membranes and Talbot et al. (2011) for feathers. Genomic DNA concentrations were quantified using fluorometry and diluted to 50 ng/mL working solutions. The sex of samples was determined using the CHD molecular sexing protocol with P8 and P2 primer pair (Griffiths et al., 1998). Genotype data were obtained from 12 autosomal nuclear microsatellite loci with the forward primer of some primers directly labeled, and some primers synthesized with an additional tail following Oetting et al. (1995), as indicated TTUCG‐2 (M13F‐29) and TTUCG‐5 (M13R‐48), Cathey et al. (1998); Aph02 (SP6 Upstream) and Aph10 (T3 Promoter), Maak et al. (2000, 2003); Bca1 (labeled), Bca7 (labeled), Bca8 (M13R‐48), Bca9 (M13R‐48), Bca11 (labeled), and Hhi3 (labeled), Buchholz et al. (1998); Smo10 (SP6 Upstream), Paulus and Tiedemann (2003); CRG (labeled), Wilson et al. (2016). For labeled primers, primers with the sequence complementary to the specific tail (or forward primer if no tail was added) were labeled with an infrared fluorophore IRD700 or IRD800 and used as the fluorescently labeled primer for detection of alleles. PCR amplification was carried out in 10 μL reaction volume with 2–100 ng of genomic DNA, 10 pM each unlabeled primer, 1 pM of IRD‐labeled primer, 0.2 mM dNTPs, 0.1 μg BSA, 1x PCR buffer (Perkin Elmer Cetus I, Norwalk, Connecticut), and 0.2 units of Taq polymerase (Promega). PCR reactions began with 94°C for 2 min followed by 40 cycles of 94°C for 15 s, 50°C for 15 s, 72°C for 30 s with a 5‐min final extension. The fluorescently labeled PCR products were electrophoresed on a 48‐well 6% polyacrylamide gel on a LI‐COR 4200LR automated sequencer (LI‐COR, Lincoln, Nebraska). Two heterozygous individuals of known size were included in all genotyping gels as size standards occupying six lanes. Ten percent of the samples were re‐extracted and genotyped in duplicate for each microsatellite for quality control purposes. Quality control checks uncovered no disparities in genotypes among 10% of the samples analyzed in duplicate. Individual samples with identical genotypes were removed from the dataset.

Nucleotide sequence data (293–294 base pairs, bp) were collected from domain 1 of the mitochondrial DNA (mtDNA) control region, using primers CI‐L78‐F and CIRH493‐R (Scribner et al., 2003). PCR amplification was carried out in 20 μL reactions with 2–100 ng genomic DNA, 10 μM each primer, 10.0 μM dNTPs, 1× PCR buffer (Perkin Elmer Cetus I), 0.1 μg BSA, and 0.5 units/μL of Taq polymerase. PCR reactions began with 94°C for 2 min followed by 40 cycles of 94°C for 15 s, 50°C for 15 s, 72°C for 30 s with a 5‐min final extension. PCR products were purified with Exo‐sap and were cycle‐sequenced via simultaneous bidirectional sequencing (SBS; LI‐COR 1999) using a commercial kit (Sequitherm LCII 2.0: Epicenter Technologies). We used fluorescently labeled universal primers (LI‐COR; M13F‐29 and M13R‐48), to prime the SBS reaction. Cycle sequencing amplification was carried out in 13 μL reactions with 1.0 pM/μL of each primer, 3.5× sequencing buffer, and 5 units/μL of Excel II polymerase. Cycle sequencing reactions began with 94°C for 2 min followed by 30 cycles of 94°C for 15 s, 50°C for 15 s, 72°C for 30 s with a 5‐min final extension. MtDNA sequences were electrophoresed on a 64‐well 3.7% polyacrylamide gel on a LI‐COR 4200LR automated sequencer and processed with eSeq and AlignIR 2.0 software (LI‐COR, Lincoln, Nebraska). GenBank accession numbers, microsatellite genotypes, and molecular sex are listed in Sonsthagen et al. (2024).

Nuclear inserts of mitochondrial DNA are common in waterfowl (Ruokonen et al., 2000; Sorenson & Fleischer, 1996). Therefore, to determine the fragment we amplified was of mitochondria in origin, we followed procedures in Sorenson and Quinn (1998) and Lanctot et al. (1999). Precautions taken to avoid amplification of nuclear inserts included amplifying tissues with low‐quality DNA such as feathers (54 samples for YKD, 42 for NS, and 77 from wintering samples not included in this study for reference mtDNA haplotypes; see Sonsthagen et al., 2024) as well as comparing sequences from mtDNA‐rich tissues such as heart muscle to blood to feathers from the same individual. In addition, sequencing gels were examined for double peaks suggestive of co‐amplification of DNA of nuclear and mtDNA origin. Sequences obtained from the same individual matched across tissue types and no ambiguous base calls were observed in the sequencing gel.

### 2.3 Selection of populations

Samples derived from molting aggregates without families occupying North Slope locales (OL, UR, UT, PB, and TESH) were initially tested against known breeders from the COL for signatures of linkage disequilibrium in GENEPOP on the Web v. 4.2 (Raymond & Rousset, 1995; Rousset, 2008) and pairwise population differentiation (*F*_ST_) at microsatellite loci in ARLEQUIN 3.1 (Excoffier et al., 2005). Samples were known to be breeders from COL as feather samples were taken from nests. After failing to observe such signatures (*p* > .05), we pooled all North Slope individuals (NS) for analyses of variance and differentiation of alleles and haplotypes, and population demography. Following similar exploratory analysis, we also combined YKD individuals (TR, BAI, KOK, KIG) into a single population.

Samples from MEL (*n* = 309) and BI (*n* = 34) were collected from individuals captured in brood drives, which comprise parent‐offspring groups but may also include molting breeders or failed breeders from other nesting locales. Use of data from brood drives may bias results by including very similar multiple first‐order relatives and (or) very dissimilar molters and failed breeders from other locales. To avoid bias, we used COLONY (Jones & Wang, 2010) to estimate genealogical relationships among the genotyped adults and goslings, based on biparentally inherited microsatellite loci only, and verified proposed parent‐offspring and sibling‐sibling relationships by (a) examining the fragment data to ensure that goslings shared at least one parental allele (at biparentally inherited loci) with each of the proposed parents or siblings, and (b) ensuring that mother‐offspring pairs shared mtDNA haplotypes. For each family group detected, we retained one sample from sibling groups and the parent(s) from parent‐offspring groupings. For genetic diversity estimates involving MEL, we include data from all individuals separated by flightless birds in remigial molt without families (MEL‐M) and known breeders that were observed with families (one representative per family group; MEL‐B). For all other analyses, we only analyzed known breeders from MEL. Final sample sizes by marker type are given in Table 1.

### 2.4 Genetic diversity

For microsatellites, we calculated allelic frequencies, expected, and observed heterozygosities, and we tested for linkage disequilibrium (LD) and deviations from Hardy–Weinberg equilibrium (HWE) using GENEPOP on the Web v. 4.2. Allelic richness was estimated using FSTAT v. 2.9.3 (Goudet, 1995, 2001). For mtDNA control region, nucleotide (*π*) and haplotype diversity (*h*) for each population was calculated in ARLEQUIN 3.1. In addition, we constructed an unrooted haplotype network for mtDNA in NETWORK 10.2.0.0 (Fluxus Technology Ltd., Clare, UK) using the median‐joining network method (Bandelt et al., 1999) to illustrate possible reticulations in the gene tree due to homoplasy or recombination. For illustrative purposes, we used the pre‐processing star contraction option (Forster et al., 2001) to decrease the number of haplotypes from 135 to 100 (see Table S1 for which samples were joined together); thus removing 35 haplotypes only observed in a single individual.

### 2.5 Population subdivision

The degree of population subdivision among locales was assessed by calculating pairwise *F*_ST_ for microsatellite loci and Φ_ST_ for mtDNA in ARLEQUIN 3.1, adjusting significant levels for multiple tests by applying Benjamini and Yekutieli‐modified false discovery rate correction (Benjamini & Yekutieli, 2001; Narum, 2006).

We determined how genetic variation is partitioned among and within sampling locales using four approaches. We first used a hierarchical analysis of molecular variance (AMOVA) in ARLEQUIN to test for significance of geographic partitioning and refugial populations proposed by Ploeger (1968) of a priori hypothesized genetic units using mtDNA and microsatellite loci with statistical significance tested by 1000 permutations. Populations were grouped to test: (a) lineage designations based on stock and subspecies (Model A); (b) the relationships between WHA brant relative to *hrota* (Model B); (c) the refugial Bering Sea region lineages, as suggested by Ploeger (1968) (Model C); (d) proposed refugia for the eastern brant (Ploeger, 1968) (Model D); (e) discreteness of WHA brant and geographic proximity of BI brant (Model E); (f) wintering region (Pacific Ocean vs. Atlantic Ocean, Model F) and MEL and PP grouped separately with *nigricans* and *hrota* populations (Models G–J). Groupings that maximize among‐group variation (Φ_CT_ or *F*_CT_) and are significantly different from random distributions are assumed to be the most probable geographical subdivisions (*p* < .05).

Secondly, we used a Bayesian‐clustering program, STRUCTURE 2.3.4 (Pritchard et al., 2000) to determine the level of population structure in the autosomal microsatellite dataset without providing a priori information on the geographic origin of the sampled individuals. STRUCTURE assigns individuals to populations maximizing Hardy–Weinberg equilibrium and minimizing linkage disequilibrium. The analysis was run for *K* = 1–10, where *K* is the number of populations using an admixture model with 100,000 burn‐in iterations and 1,000,000 Markov chain Montel Carlo (MCMC) iterations. The analysis was repeated 10 times for each *K*. We used the Δ*K* method of Evanno et al. (2005) and evaluated the estimate of the posterior probability given *K*, Ln *p*(*D*), to determine the most likely number of groups at the uppermost level of population structure. Wang (2017) showed STRUCTURE can yield poor individual assignment when there is uneven sampling across the proposed source populations (*K*). Following the recommendations of Wang (2017) we explored alternative ancestry priors (ALPHA value) that allows for unequal representation of the source populations by the dataset as well as using the correlated frequency model. The results were summarized in STRUCTURE HARVESTER web v.0.6.94 (Earl & vonHoldt, 2012). Final assignment probabilities were based on the optimal clustering alignment across all 10 replicates for the optimum *K* using the GreedySearch algorithm for 1000 iterations in the program CLUMPP v.1.1 (Jakobsson & Rosenberg, 2007). Lastly, population structure was estimated using a principal components analysis (PCA) as implemented by the package adegenet (Dray & Dufour, 2007; Jombart, 2008) in the program R 4.0.1 (R Core Team, 2020). For PCA, we plotted only the first two principal components.

### 2.6 Demographic history

To test for genetic signatures of recent effective population size changes based on mtDNA sequence data, we calculated Fu's *F*_S_ (Fu, 1997) and Tajima's *D* (Tajima, 1989) in ARLEQUIN. Negative values of either statistic result when there is an excess of low‐frequency polymorphisms which can result from rapid population expansion or selective sweep acting on linked polymorphism. Conversely, a positive value can be indicative of population decline.

For microsatellites, we assessed evidence for fluctuations in population size by using BOTTLENECK 1.2.02 (Cornuet & Luikart, 1996). BOTTLENECK compares the number of alleles and gene diversity at polymorphic loci under the infinite‐allele (IAM; Maruyama & Fuerst, 1985), stepwise mutation model (SMM; Ohta & Kimura, 1973), and two‐phase model of mutation (TPM; Di Rienzo et al., 1994). Parameters for the TPM were set at 79% SMM with a variance of 9% (Garza & Williamson, 2001; Piry et al., 1999), with 5000 simulations performed for each population. Significance was assessed using a Wilcoxon sign‐rank test, which determines whether the average of standardized differences between observed and expected heterozygosity is significantly different from zero (Cornuet & Luikart, 1996). Significant heterozygote deficiency relative to the number of alleles indicates recent population growth, whereas heterozygote excess relative to the number of alleles indicates a recent population bottleneck (Cornuet & Luikart, 1996).

### 2.7 Analysis of gene flow

To understand levels and directionality of gene flow between and among North American regions, the number of migrants per generation (*N*_*e*_ *m* or *N*_*f*_ *m*) among seven sampled localities was calculated for nuclear microsatellite loci and mtDNA in MIGRATE v3.0.3 (Beerli & Felsenstein, 1999, 2001). The following sampling localities were used: (a) Prince Patrick Island and (b) Melville Island (breeders only) were used to represent the Western High Arctic, (c) Colville, Alaska (known breeders) was used to represent the North Slope, (d) Kigigak Island, Alaska represented the YKD, (e) Baffin Island and (f) Southampton Island represented populations that migrate along the Atlantic coast, and (g) Bathurst Island represented the Eastern High Arctic. Palearctic locales were not used in this analysis due to low sample size. Full models, *θ* (4*N*_*e*_μ or *N*_*f*_μ), and all pairwise migration parameters were allowed to vary and estimated individually from the data and compared to restricted island models for which *θ* and pairwise migration parameters were equal among populations (symmetrical gene flow). For microsatellites, MIGRATE was run using maximum likelihood search parameters; 10 short chains (500 out of 100,000 sampled trees), five long chains (7500 out of 1500,000 sampled trees), and five adaptively heated chains (start temperatures 1, 1.5, 3, 6, and 12; swapping interval = 1) with burn‐in 4,000,000 per chain. For mtDNA, MIGRATE was run using maximum likelihood search parameters; 10 short chains (2500 out of 625,000 sampled trees), five long chains (10,000 out of 2500,000 sampled trees), and five adaptively heated chains (start temperatures 1, 1.5, 3, 6, and 12; swapping interval = 1) with burn‐in 12,500,000 per chain. Full models and restricted models were run five times to ensure the convergence of parameter estimates. Alternative models were evaluated for goodness of fit given the data using a log‐likelihood ratio test. The resulting statistic from the log‐likelihood ratio test is equivalent to a *χ* ^2^ distribution with the degrees of freedom equal to the difference in the number of parameters estimated in the two models (Beerli & Felsenstein, 2001).

## 3 RESULTS

### 3.1 Genetic diversity

The average number of alleles per population varied from 2.9 (IRE) to 6.9 (LRD); allelic richness ranged from 2.7 (SH, BAF, BI) to 3.2 (YKD); observed heterozygosity ranged from 33.2% to 42.2% (Table 1), averaging 38.4% overall. No locus (*χ* ^2^ = 0.0–47.8, df = 10–26, *p* > .0038) deviated from HWE except for Smo10, which deviated from HWE in both MEL‐B and MEL‐M (*p* < .0007). No population deviated from HWE overall (*χ* ^2^ = 3.5–38.5, df = 16–22, *p* > .07). LD was not observed for any locus pair overall (*χ* ^2^ = 3.5–38.4, df = 10–26, *p* > .056) except for 5AB and Aph02 (*χ* ^2^ > 45.3, *p* < .002) due to LD at that locus pair in one population (NS, *p* < .05). Allelic richness and observed heterozygosity were higher in locales occupied by *nigricans* than *hrota* (Table 1).

One hundred thirty‐five unique mtDNA haplotypes based on 51 variable sites (37 parsimony informative sites) were identified from 386 individuals (Table S2). Haplotype (*h*) and nucleotide (*π*) diversity ranged from 0.644 to 0.992 and 0.0095 to 0.0394, respectively (Table 1). The highest *h* diversity estimates were observed for the WHA group (average *h* = 0.951) and the lowest observed for *nigricans* (average *h* = 0.834), among which YKD demonstrated the lowest *h* and *π* for nesting areas.

**FIGURE 3:**
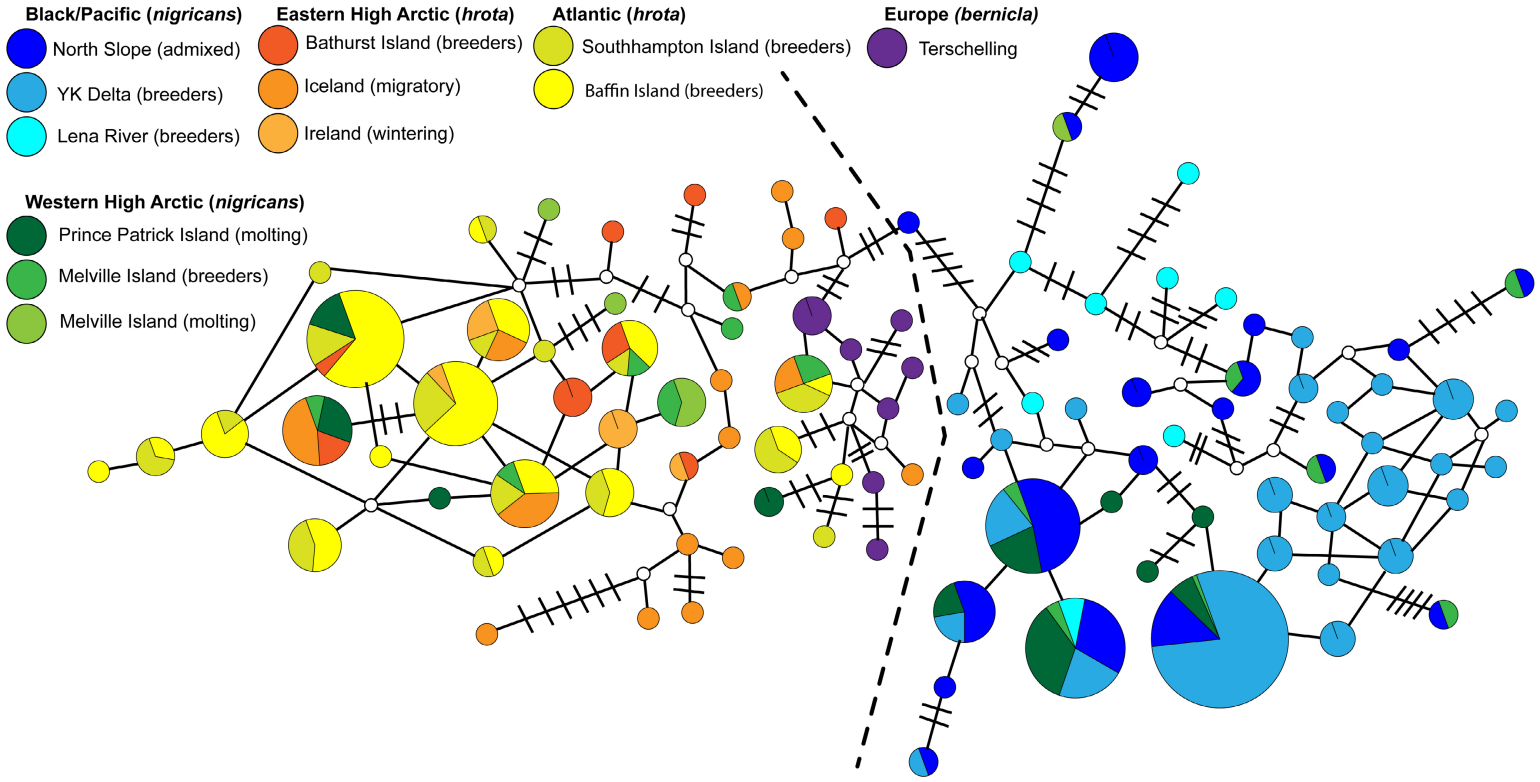
Unrooted haplotype network illustrating relationships of 130 mtDNA control region haplotypes assayed (star contraction option reduced the 135 haplotypes to 100; see Table S1) from brant (*Branta bernicla*). The size of the circle corresponds to the frequency of each haplotype and small white circles indicate intermediate ancestral haplotypes that were not sampled. As branch lengths are not to scale, the number of marks across branches indicate the number of variable sites between haplotypes. No marks indicate only a single variable site between haplotypes. The dashed line separates haplotypes of *B. b. nigricans* and *B. b. hrota*.

An unrooted network based on the mtDNA control region (Figure 3) placed brant haplotypes assayed from North America into two main clusters: an Atlantic (east) cluster and a Pacific (west) cluster. No haplotypes from *nigricans* populations were placed in the Atlantic cluster, and no haplotype from the *hrota* was placed into the Pacific cluster. In addition, haplotypes from the western arctic Russia (represented by wintering birds from Terschelling Island) were placed in the Atlantic cluster while haplotypes from Lena River, in the central arctic Russia were within the Pacific haplotype cluster. Haplotypes found on Melville and Prince Patrick islands (WHA) occurred in both clusters with the majority from Prince Patrick Island in the Pacific (west) and a majority from Melville Island within the Atlantic (east).

### 3.2 Population subdivision

Overall estimates of population subdivision were significant for both microsatellite loci (*F*_ST_ = 0.068, *p* < .001) and mtDNA (Φ_ST_ = 0.691, *p* < .001). Significant estimates of inter‐population variance in allelic frequency were observed among, not within, subspecies (*F*_ST_ = −0.015–0.094; Table 2); most significant pairwise *F*_ST_ values involved comparisons of *nigricans* populations or *bernicla* (TER) with populations (e.g., BAF and SH) ascribed to *hrota* (Table 2). In contrast to nuclear loci, very high levels of inter‐population variance in mtDNA haplotypic frequency were observed among most population pairs (Φ_ST_ = 0.028–0.830; Table 2), with nearly all the populations demonstrating significant differentiation (*p* < .00876, Table 2).

**TABLE 2:** Results of pairwise tests for population differentiation based on variance in allele frequency: *F*_ST_ based on microsatellite data are below the diagonal and Φ_ST_ based on mtDNA control region are above the diagonal with shaded comparisons indicating comparison is within same migratory pathway.

	Atlantic		Eastern high Arctic			Pacific Black				Western high Arctic		Europe
	BAF	SH	IRE	ICE	BI	LB	NS	YKD	LRD	MEL‐B	PP	TER
BAF	‐	0.028	0.106	**0.157**	**0.194**	‐	**0.685**	**0.830**	**0.701**	**0.400**	**0.567**	**0.614**
SH	−0.004	‐	0.111	**0.090**	**0.119**	‐	**0.621**	**0.807**	**0.584**	**0.263**	**0.466**	**0.432**
IRE	−0.010	0.000	‐	0.105	**0.182**	‐	**0.626**	**0.830**	**0.623**	**0.189**	**0.447**	**0.667**
ICE	0.002	0.003	−0.012	‐	0.076	‐	**0.546**	**0.764**	**0.474**	**0.057**	**0.380**	**0.366**
BI	0.007	0.013	0.007	0.001	‐	‐	**0.580**	**0.801**	**0.549**	**0.170**	**0.408**	**0.541**
LB	**0.091**	**0.094**	**0.064**	**0.072**	0.096	‐	‐	‐	‐	‐	‐	‐
NS	**0.073**	**0.074**	**0.050**	**0.064**	**0.091**	−0.001	‐	**0.163**	0.113	**0.480**	**0.078**	**0.559**
YKD	**0.069**	**0.072**	**0.046**	**0.058**	**0.079**	−0.005	0.000	‐	**0.507**	**0.756**	**0.341**	**0.799**
LRD	**0.085**	**0.094**	0.053	**0.075**	**0.092**	0.014	0.015	0.011	‐	0.363	0.105	**0.509**
MEL‐B	**0.015**	**0.015**	0.003	0.004	0.024	**0.029**	**0.028**	**0.025**	**0.047**	‐	**0.285**	**0.461**
PP	**0.046**	**0.048**	0.019	**0.033**	**0.064**	0.011	**0.008**	**0.006**	0.023	0.008	‐	**0.420**
TER	**0.039**	**0.039**	0.023	**0.034**	**0.054**	0.12	0.012	0.044	0.019	0.012	0.009	‐

*Note*: Pairwise estimates are provided for each pair of 12 sampling locations in North America and Russia. Significant values are in bold after Benjamini and Yekutieli‐modified false discovery rate correction (*p* < .0087; 169 comparisons). Population abbreviations and locality are defined in Section 2 and Table 1 and Figure 1, respectively.

The *F*_CT_ maximized by the AMOVA revealed similar genetic partitions based on microsatellite and mtDNA data (Table 3). The current subspecies/stock hypothesis (Model A, Table 3) did not account for the highest among‐group variation for estimates based on microsatellite (*F*_CT_ = 0.045) and mtDNA (*F*_CT_ = 0.549). Rather, *F*_CT_ was maximized for microsatellite variation when WHA brant are split between *nigricans* [PP] and *hrota* [MEL] (Model G, *F*_CT_ = 0.056) whereas variation at mtDNA was maximized when populations were grouped by wintering areas (Model F, *F*_CT_ = 0.599). Although Model G and Model F explained the highest amount of variation, there were several models that had similarly high values of *F*_CT_ (see Table 3).

**TABLE 3:** Hierarchical analyses of variance for hypothesized groupings, based on fragment data from 12 autosomal microsatellite loci (top) and mtDNA sequence data (bottom).

Model	Hypothesized groupings	Variance components		
		Among individuals	Among populations	Among groups
Microsatellite loci				
A: Subspecies/stock	[SH, BAF] [ICE, BI, IRE] [PP, MEL] [YKD, NS, LB, LRD]	**0.046**	0.001	**0.045**
B: WHA with *hrota*	[SH, BAF, PP, MEL] [ICE, BI, IRE] [YKD, NS, LB, LRD]	**0.047**	**0.010**	0.037
C: WHA with *nigricans*	[SH, BAF] [ICE, BI, IRE] [PP, MEL, YKD, NS, LB, LRD]	**0.055**	**0.006**	**0.049**
D: EHA with *hrota*	[SH, BAF, ICE, BI, IRE] [PP, MEL] [YKD, NS, LB, LRD]	**0.050**	0.002	**0.048**
E: WHA with BI	[SH, BAF, ICE, IRE] [PP, MEL, BI] [YKD, NS, LB, LRD]	**0.048**	**0.004**	**0.044**
F: Atlantic and Pacific	[SH, BAF, ICE, BI, IRE] [PP, MEL, YKD, NS, LB, LRD]	**0.061**	**0.006**	**0.055**
G: MEL with Atlantic	[SH, BAF, ICE, BI, IRE, MEL] [PP, YKD, NS, LB, LRD]	**0.060**	**0.004**	**0.056**
H: PP with Atlantic	[SH, BAF, ICE, BI, IRE, PP] [MEL, YKD, NS, LB, LRD]	**0.052**	**0.014**	0.039
I: MEL with EHA	[SH, BAF] [ICE, BI, IRE, MEL] [PP, YKD, NS, LB, LRD]	**0.052**	0.003	**0.049**
J: PP with EHA	[SH, BAF] [ICE, BI, IRE, PP] [MEL, YKD, NS, LB, LRD]	**0.047**	0.012	0.035
MtDNA control region				
A: Subspecies/stock	[SH, BAF] [ICE, BI, IRE] [PP, MEL] [YKD, NS, LRD]	**0.616**	**0.147**	**0.549**
B: WHA with *hrota*	[SH, BAF, PP, MEL] [ICE, BI, IRE] [YKD, NS, LRD]	**0.620**	**0.307**	**0.452**
C: WHA with *nigricans*	[SH, BAF] [ICE, BI, IRE] [PP, MEL, YKD, NS, LRD]	**0.658**	**0.210**	**0.568**
D: EHA with *hrota*	[SH, BAF, ICE, BI, IRE] [PP, MEL] [YKD, NS, LRD]	**0.634**	**0.149**	**0.570**
E: WHA with BI	[SH, BAF, ICE, IRE] [PP, MEL, BI] [YKD, NS, LRD]	**0.626**	**0.192**	**0.537**
F: Atlantic and Pacific	[SH, BAF, ICE, BI, IRE] [PP, MEL, YKD, NS, LRD]	**0.681**	**0.204**	**0.599**
G: MEL with Atlantic	[SH, BAF, ICE, BI, IRE, MEL] [PP, YKD, NS, LRD]	**0.672**	**0.189**	**0.597**
H: PP with Atlantic	[SH, BAF, ICE, BI, IRE, PP] [MEL, YKD, NS, LRD]	**0.647**	**0.324**	**0.478**
I: MEL with EHA	[SH, BAF] [ICE, BI, IRE, MEL] [PP, YKD, NS, LRD]	**0.615**	**0.311**	0.441
J: PP with EHA	[SH, BAF] [ICE, BI, IRE, PP] [MEL, YKD, NS, LRD]	**0.622**	**0.293**	**0.465**

*Note*: Fixation indices are shown and the proportion of the total variance that is explained by the hypothesized regional grouping is the among‐groups value. Values in bold text are significantly different from zero (*p* < .05). Population abbreviations and locality are defined in Section 2 and Table 1.

STRUCTURE analysis indicated the most likely number of genetic clusters was two based on both Evanno's method (*K* = 2; Δ*K* = 544.5 vs. *K* = 4; Δ*K* = 9.11) and LN *p*(*K*) (*K* = 2; −12,706 SD 1.56 vs. *K* = 1; −13,124 SD 0.05 vs. *K* = 4; −13,080 SD 84.60; Figure S1). Using alternative ancestry priors (ALPHA value) and the correlated frequency model produced the same result of *K* = 2 being the most likely number of clusters. As detected in the mtDNA network, the two groups correspond to an Atlantic (east) cluster and Pacific (west) cluster (Figure 2). There were a number of samples from both groupings with intermediate assignment probability (<75%) to either group, potentially representing admixed ancestry (Table S3). The Western High Arctic contained the highest proportion of samples with intermediate assignment probability (30%–39%) and samples assigned to both groups (17%–44%) based on an assignment probability of >75% to one cluster. Admixed ancestry was also observed in samples from all locales except Bathurst Island which were all assigned to the Atlantic cluster. Individuals placed within a non‐origin grouping within North American sampling locations ranged from 0% to 12%. No additional structure was detected when analyzing the two clusters separately. For *K* = 4, the majority of samples were not assigned to a specific cluster (<75%; Figure S2). However, two samples from TER (*bernicla*) and nine of 11 samples from *hrota* that are known to potentially migrate to Europe (4 ICE, 1 IRE, 1 SH, 1 MEL‐B, 1 MEL‐M, and 1 PP) were assigned to a cluster with >0.80%.

**FIGURE 4:**
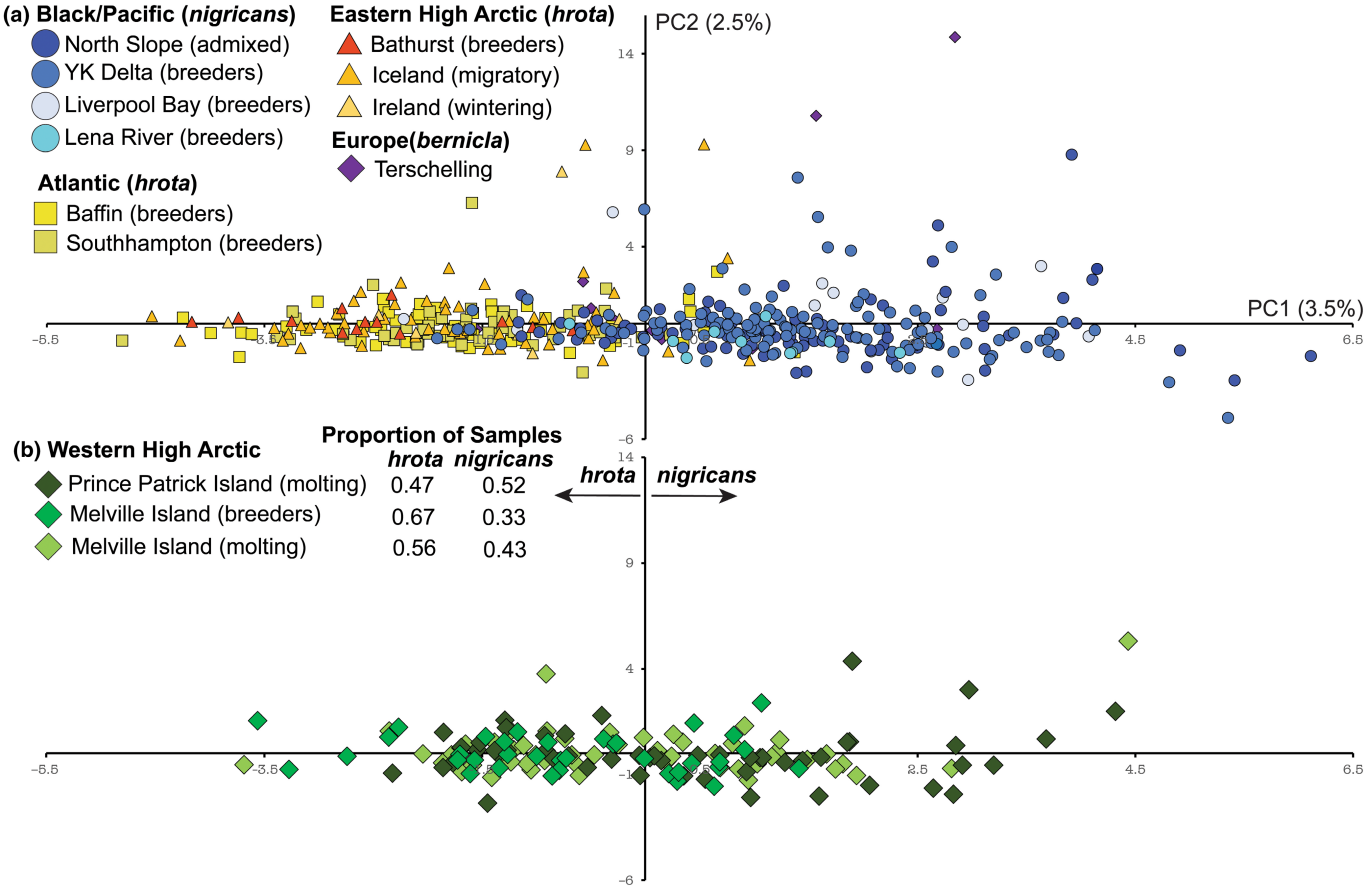
PCA plot of first two principal components based on 12 microsatellite loci. For illustrative purposes, Western High Arctic samples were not shown in the (a) top panel but are shown in (b) bottom panel. Both panels represent the same analysis. Although there is some overlap, the *x*‐axis divides the majority of *nigricans*‐like (Pacific/Black) and *hrota*‐like (Atlantic/EHA) genotypes. The table in the bottom panel reports the proportion of samples from the Western High Arctic on either side of the *y*‐axis (positive or negative PC1 coordinate value).

The PCA was congruent with the STRUCTURE analysis with samples aligning mainly within the Atlantic and Pacific groups with some overlap (Figure 4). Melville Island and Prince Patrick Island samples (WHA) were observed within each main grouping with a higher percentage of Melville Island individuals in the Atlantic group and higher percentage of Prince Patrick Island individuals in the Pacific group.

### 3.3 Demography history

Significant fluctuations in population demography were indicated based on both marker types in multiple populations (Table 1). All Atlantic *hrota* (BAF and SH), one EHA *hrota* (ICE), three *nigricans* (NS, LB, YKD), and one WHA (PP) populations showed heterozygote deficiency at microsatellite loci, suggestive of recent population growth or nonrandom mating, under the SMM. Remaining populations were in mutation‐drift equilibrium across all tests.

Population growth based on mtDNA sequence data was indicated for breeding *hrota* populations BAF and SH and the staging ICE *hrota* populations based on at least one of the metrics that estimate population change from sequence data (Table 1). Within *nigricans*, breeding populations YKD and MEL (WHA) local breeders showed a signature of population growth or expansion (Fu's *F*_S_, Table 1).

### 3.4 Gene flow

**FIGURE 5:**
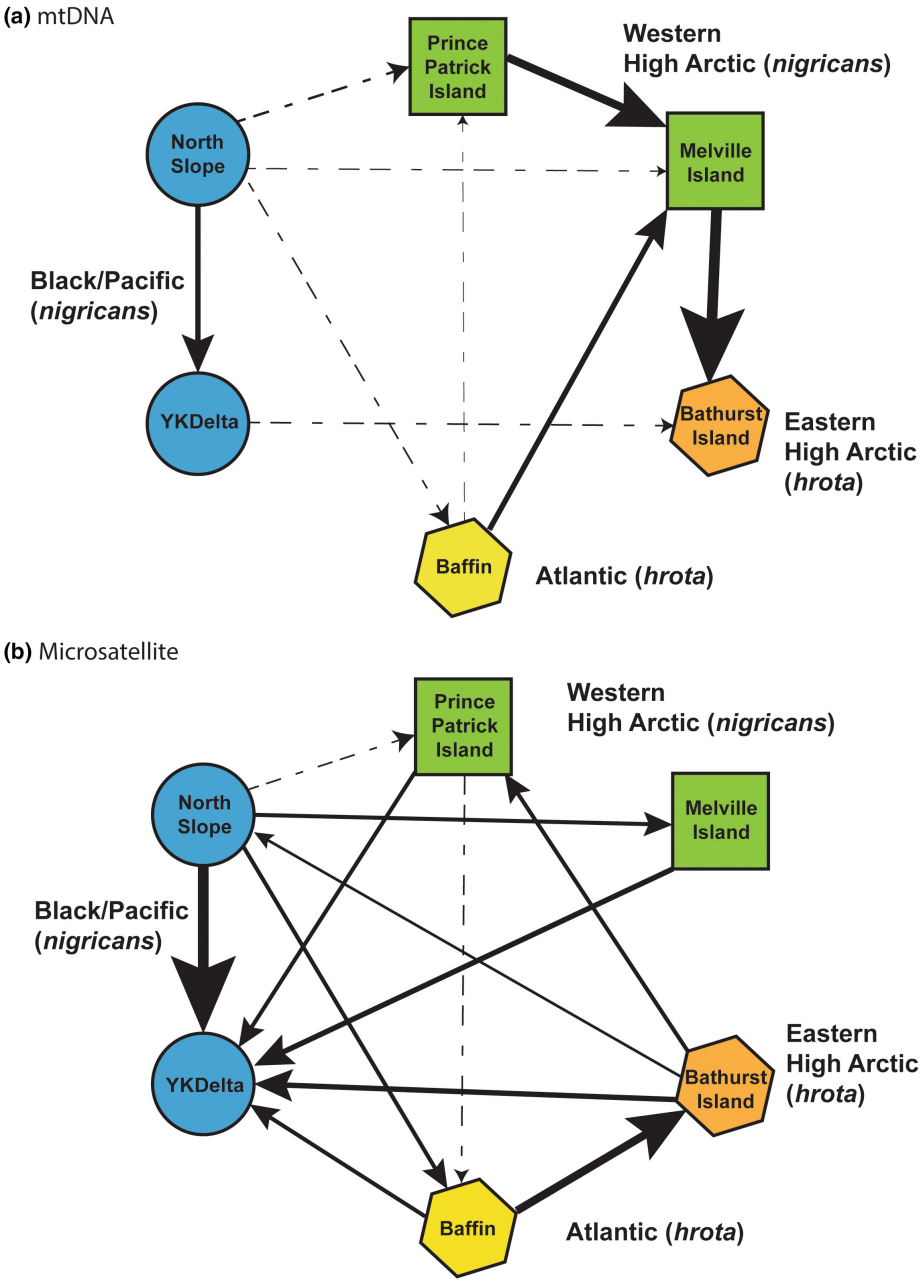
Results of full model migration matrix when parameters were allowed to vary independently to estimate gene flow among brant populations in North America. Migration direction and rates are based on (a) mtDNA control region data and (b) 12 microsatellite loci. Arrow thickness is proportionate to estimated levels of gene flow (thicker arrows indicate higher relative gene flow). Solid lines indicate 95% confidence intervals (CI) did not overlap while dashed lines indicate 95% CI overlap but did not encompass either mean value. No lines indicate either symmetrical gene flow or no gene flow between populations. See Table S4 for values.

Gene flow estimates based on both marker classes indicated gene flow among populations was generally higher within subspecies or region than among regions/subspecies (Figure 5; Table S4). In general, the North Slope (represented by Colville) was a net exporter of individuals to most other regions and there was a generally west to east direction of gene flow in mtDNA. There were low number of female migrants per generation for mtDNA (*N*_*f*_ *m*) detected into the North Slope or YKD (*nigricans*) from *hrota* locales (0.1–0.3) while 0.2–4.2 were calculated going into the *hrota* locales from the Pacific region (Table S4). For microsatellites, North Slope was an exporter of individuals into Western High Arctic and Baffin indicated west to east directionality. The YKD was a net receiver of individuals from all locales in the Pacific, Western High Arctic, and Eastern High Arctic while North Slope was a net importer from Bathurst Island, suggestive of an east to west directionality.

## 4 DISCUSSION

The geographic patterns of genetic structure we observed across the brant's North American range conformed at least partially to phenotypic characteristics and migratory routes. There was an east–west partition in both marker types, although more discrete in mtDNA. This is consistent with migratory routes detected using band recovery and telemetry data (Boyd et al., 2013; Castelli et al., 2010; Erskine, 1988; Leach et al., 2018; Pennycuick et al., 2011). In addition, this genetic partition corresponds with the described phenotypically distinct subspecies in the west (*nigricans*) and in the east (*hrota*). The phenotypically intermediate WHA brant did not form a distinct genetic group but rather contained haplotypes observed on either side of the migratory divide as well as a variable degree of mixed ancestry in the microsatellite dataset. In contrast to Shields (1990), who suggested this population likely diverged in isolation, our data suggest the intermediate coloration evolved due to the formation of a historical contact zone and has potentially been maintained via contemporary hybridization.

Outside of North America, the Lena River delta samples were grouped within *nigricans* where birds from the central and eastern portion of this delta are placed based on plumage (belly color) and migration routes east to the Pacific region (Sawa et al., 2019, 2020). However, our sample of wintering brant at Terschelling Island, the Netherlands (*bernicla*) showed considerable admixture with microsatellite data and mtDNA haplotypes were placed within *hrota* haplotypes. The wintering sample from Terschelling Island showed a similar pattern of admixture as the WHA (Melville and Prince Patrick islands) samples. A genomic approach may be needed to determine whether the different phenotypes are maintained through ongoing gene flow across the Western High Arctic in North America or through wintering sites in Europe or if admixed ancestry is the result of a historical introgression event with subsequent isolation where plumage differences are maintained through subtle genetic differences. Overall, our results indicate that both historical and contemporary processes have shaped the distribution of genetic variation among populations.

### 4.1 Maintenance of regional structure in North America

We found significant Atlantic (east)–Pacific (west) differentiation based on mtDNA control region sequence and microsatellite fragment data within North American brant. Of note is that no regional haplotypes are shared across this migratory divide which suggest there is no or very restricted movement across these two broad regions, except within the Western High Arctic (Parry Islands). Furthermore, waterfowl are often characterized by male‐biased dispersal which is most often the mechanism implicated for the lack of genetic structure (Brown et al., 2020; Sonsthagen et al., 2019; Wilson et al., 2016). Our dataset contains approximately 27% males (Ratio Male:Female:Unknown 1.0:1.8:0.9) and given the higher propensity for male dispersal in brant (Lindberg et al., 1998), we would have expected to see at least a low level of haplotype sharing across regions if there were no dispersal restrictions (e.g., behavioral or geographical) even though the paternal mtDNA haplotype is not inherited (see Brown et al., 2020). This is further suggestive of long‐term isolation based on female samples and limited short‐term movement within the male cohort sampled, which corroborates telemetry and banding data (Castelli et al., 2010).

The observed patterns of spatial variance at microsatellite loci and mtDNA sequence data within North American brant in our study differ from other geese with similar nesting distribution across the North American Arctic (Wilson et al., 2018). For example, eastern and western regions of greater white‐fronted geese share high‐frequency (i.e., common) mtDNA haplotypes while low‐frequency haplotypes are typically not shared (Wilson et al., 2018), which is also observed in the snow goose (*Anser caerulescens*; Shorey, 2005). Differences in the level of genetic structure across these species are likely not only due to the length of isolation but a combination of other mechanisms. Genetic structure in greater white‐fronted geese is likely influenced by the timing of events in the annual cycle limiting availability of potential mates, which maintains subtle regional genetic differentiation (Wilson et al., 2022). Shifts in migration ranges accompanied the significant population increase in snow geese (Alisauskas et al., 2022), which may facilitate changes in genetic structure when formerly separate populations come into contact. In contrast, the genetic structure observed in this study of North American brant is likely maintained by disjunct distributions across the annual cycle (Lewis et al., 2020) which restricts potential inter‐regional mating opportunities. The distinct regional separation in brant, while other species exhibit signatures of admixture, may be attributable to the relatively stable or declining population trends experienced in brant (Olson, 2021; Roberts et al., 2021; Sedinger et al., 2019) such that population densities have not changed sufficiently to influence dispersal decisions as in snow geese.

### 4.2 Contact zone connectivity

The genetic uniqueness of WHA brant (Parry Islands) is supported by the presence of mtDNA haplotypes from both the west (*nigricans*) and east (*hrota*) as well as individuals with intermediate assignment probabilities (in approximately equal proportion with high assignment to the two genetic clusters) based on the microsatellite data (see Table S3). The pattern of admixture contrasts with Pacific black brant and Atlantic/EHA individuals which had relatively high assignment probabilities to their region of origin and low proportion of admixed individuals. A greater proportion of individuals within the Pacific groupings had mixed ancestry or assignments to the Atlantic/EHA genetic cluster. The asymmetrical pattern in the presence of mixed ancestry and Atlantic genetic‐affiliated individuals in the Pacific region may be due, at least in part, to differences in annual movement patterns among the four brant nesting in Pacific, Atlantic, Eastern High Arctic, and the Western High Arctic areas which either facilitate or limit opportunities for intermixing.

Telemetry and mtDNA data suggest limited direct contact between populations wintering on the Pacific and Atlantic coasts. However, the microsatellite data indicate that *nigricans* and *hrota* brant are still connected, as brant assigned to genetic clusters affiliated with the different subspecies as well as admixed brant were observed in both Pacific and Atlantic regions (see STRUCTURE analysis). Furthermore, there was a relatively high proportion of breeding individuals with admixed or high assignment to the non‐origin genetic cluster in the Pacific region with highest observed in the YKD area. Failed or nonbreeding Pacific brant may undergo long (>1500 km) molt migrations that are not typical of the other brant stocks (Lewis et al., 2020). The largest of these molting concentrations occur on the North Slope of Alaska and comprises individuals from several breeding colonies originating in Alaska and Canada (Bollinger & Derksen, 1996). In contrast, Atlantic and Eastern High Arctic brant molt locally in areas that are nearer to breeding sites (Lewis et al., 2020), which may limit groupings with individuals from other nesting colonies and intermixing of populations on wintering areas. Difference in molt migration patterns could explains the low percentage of individuals assigned an intermediate (0%–19%) or a non‐origin region (0%–2%) for Atlantic brant based on microsatellite data. The known breeders within the Western High Arctic (Melville Island) were predominately assigned to Atlantic/east grouping (39%) or an admixed group (44%) while molting individuals (Melville and Prince Patrick islands) were equally assigned to all three groups (*hrota*, *nigricans*, or admixed). The presence of individuals assigned to all three groups, in roughly equal proportions, could be explained by the large molting concentrations of brant from multiple colonies in the Western High Arctic (i.e., Banks Island; Cotter & Hines, 2001). Further, brant from the Parry Islands (WHA) predominately stage in the fall and winter in areas used by Pacific *nigricans* (Boyd et al., 2013; Reed, Stehn, et al., 1989) and appear less likely to utilize wintering areas occupied by Atlantic or Eastern High Arctic brant, despite being in closer geographic proximity to those breeding colonies (e.g., Bathurst Island).

As most pair bonds are likely formed at various stages during the annual cycle outside of breeding areas (Lewis et al., 2020; Lindberg et al., 1998), the higher proportion of admixture observed within Pacific brant is likely maintained by sympatry between Pacific and WHA brant during certain periods of the annual cycle. In comparison, banding and telemetry data for greater white‐fronted geese did not detect dispersal events between Alaska and Asia (Wilson et al., 2018); however, genomic data showed that dispersal does occur albeit very infrequently (Wilson et al., 2022). As banding data are used to evaluate general migratory patterns rather than individual movements, the admixed proportion of individuals in the Eastern High Arctic and Atlantic brant could be maintained through low levels of across‐continent dispersal as has been observed in greater white‐fronted geese as opposed to being mediated through a contact zone or retention of ancestral lineages. The same mechanism could promote admixture between *bernicla* and *hrota* in Europe. Although *hrota* primarily breeds in North America, there are small nesting populations in Svalbard, Franz Josef Land, and northwestern Greenland that winter in western Europe where *hrota* intermixes with *bernicla* (Koffijberg et al., 2013). Moreover, *hrota* from eastern Canada migrate over Iceland to winter in Ireland and also mix with *bernicla* on the Channel Islands (UK) and in Normandy (Dalloyau, 2022; Lewis et al., 2020). In the STRUCTURE analysis, five of the 13 brant from Terschelling Island (all classified by plumage characteristics as *bernicla*) were assigned to *hrota* at *K* = 2. However, when *K* = 4, two samples were placed in a separate group (also indicated in PCA analysis) with five *hrota* from Iceland or Ireland and four nonbreeding individuals from WHA. Grouping of individuals ascribed to *bernicla*, *hrota*, and to a lower extent *nigricans* suggests that inter‐continental gene flow could be maintained via intermixing on wintering grounds in Europe as has been observed for WHA based on banding data (Boyd et al., 1988; Boyd & Maltby, 1979). However, disentangling these alternatives (indirect vs. direct connectivity) would require additional genomic analysis, a larger sample size, and samples from nesting populations of *bernicla*.

### 4.3 Demographic history

Geographic genetic partitions in North America brant are also likely influenced by Pleistocene events associated with glacial refugia and population expansion following climate amelioration. During the Last Glacial Maximum, glacial ice sheets covered most of northern North America except for areas in Alaska and along the Pacific and Atlantic coasts (Hewitt, 2001, 2004). This may have isolated brant populations into western and eastern refugia, promoting the partitioning of genetic variation detected in both mtDNA and microsatellites in the AMOVA analysis. A strong east–west divide, whether observed in migration routes or genetic structure, is common in bird species including other Arctic species (e.g., common eider, *Somateria mollissima*, Sonsthagen et al., 2011; snow bunting, *Plectrophenax nivalis*, Macdonald et al., 2012).

As brant nest predominantly in the Arctic, Ploeger (1968) hypothesized that, across its distribution, the species likely shifted nesting grounds to unglaciated tundra habitats exposed during the maximum of eustatic lowering of sea level, rather than to low Arctic conditions found southward. Considering the morphological differences between the light‐bellied subspecies (*hrota*) and the black‐bellied forms (*nigricans*), Ploeger (1968) suggested that the ancestors of *hrota* were isolated in a refugium in the North Atlantic region (likely in western Europe), and the black‐bellied forms in eastern Siberia and the Bering Sea region (Beringia). Ancestors of *hrota* could have spread northward, then westward into the Canadian Arctic, meeting black‐bellied forms spreading out of the eastern Siberia/Beringian refugium (Ploeger, 1968), a hypothesis supported by our genetic data.

Phylogeographic studies of terrestrial and freshwater species in northern high latitudes have identified a series of genetic discontinuities common across multiple taxa, hypothesized to have resulted from the retention and isolation of lineages in different refugia. In North America, discontinuities in terrestrial plants and mammals have been described at the Parry Channel and the Mackenzie River in Northern Canada (Abbott et al., 2000; Fedorov et al., 2003; Fedorov & Stenseth, 2002; MacPherson, 1965; Tremblay & Schoen, 1999). Discontinuities between populations on islands to the north and east of the Parry Channel and Canadian low Arctic populations (Arctic dryad, *Dryas integrifolia*, Tremblay & Schoen, 1999; collared lemming, *Dicrostonyx groenlandicus*, Fedorov et al., 1999) signal retention of isolated lineages in a Canadian high Arctic refugium (MacPherson, 1965; Pielou, 1991).

### 4.4 Implications for management and conservation

Brant in North America have undergone wide fluctuations in population numbers beginning early in the 20th century (Olson, 2021). During the 1930s, a wasting disease associated with the heterokont *Labyrinthula zosterae* caused extensive losses of eelgrass, important forage for brant, on both coasts of North America, although more extensively on the Atlantic coast (Cottam et al., 1944; Rogers, 1979). During the 1970s and 1980s, population numbers of brant nesting and wintering in Pacific locales in North America declined markedly, largely due to reductions in the nesting populations on the YKD during the 1970s and 1980s (Sedinger et al., 1994). Although numbers have relatively stabilized on both coasts (Lewis et al., 2020), brant populations have not significantly increased in size as have other Arctic geese species starting in the late 1980s (Olson, 2021). Reductions in the size of breeding populations may result in corresponding decreases in genetic diversity, and thus brant became a species of increased concern in the latter part of the 20th century (Sedinger et al., 1994). Brant nesting in the YKD showed signatures of population growth at both marker types and have similar levels of genetic diversity as other areas throughout the species range. However, rapid recovery of bottlenecked populations is not always reflected in a loss of genetic diversity (Busch et al., 2008), but prolonged reductions in the number of individuals can reduce genetic variation due to inherent time lag between population demography and resulting genetic consequences (Epps & Keyghobadi, 2015). Because this species is characterized as having low reproductive rates and thus recruitment (Lewis et al., 2020), the continual recording of temporal changes in genetic diversity is still needed. This is important because changes in genetic structure in terms of allele richness and heterozygosity levels can yield changes in individual fitness components (i.e., genetic Allee effect) over a different timescale making its effect not as obvious as an ecological factor (Luque et al., 2016).

Our results based primarily on mtDNA reveal that the Atlantic and Pacific Flyway populations are genetically discrete, and the Western High Arctic populations nesting on Melville and Prince Patrick islands likely represent a secondary contact zone between the two lineages, and thus form a “zone of intergradation” (Corbin, 1987) between *hrota* and *nigricans*. Although individuals from the Western High Arctic do not constitute a genetically distinct lineage based on our analyses, the region likely had a large influence on the overall population dynamics and maintenance of genetic diversity within North America despite the small population size. Given the potential of brant for low reproductive output during certain years (Sedinger et al., 2006), the resiliency and short‐term response of populations may be tied to the maintenance of individual phenotypic plasticity which is linked to genetic variation in “construction” traits (e.g., movement, habitat preferences; Shaw, 2020; Snell‐Rood & Steck, 2019). Although our study did not assess functional genes, the presence of high genetic diversity and unique movement types in the Parry Islands (WHA) brant suggests that they may act as a reservoir of genetic and behavioral diversity and therefore may represent an area of high conservation value for the persistence of brant through this time of rapid change. Similarly, our data suggest the potential for inter‐continental connectivity between North America *hrota* and European *bernicla* wintering groups. Additional studies to determine whether certain nesting areas not sampled in this study for *hrota* (e.g., Greenland) and for *bernicla* (e.g., western Lena River and Olenyok River deltas) are more likely promoting gene flow across subspecies would add to our knowledge on the dispersal and genetic structure of these regions.

## AUTHOR CONTRIBUTIONS

**Robert E. Wilson:** Data curation (equal); formal analysis (lead); investigation (equal); writing – original draft (lead). **W. Sean Boyd:** Conceptualization (equal); funding acquisition (equal); resources (equal); writing – review and editing (equal). **Sarah A. Sonsthagen:** Data curation (equal); formal analysis (supporting); investigation (equal); writing – original draft (supporting). **David H. Ward:** Conceptualization (equal); funding acquisition (equal); resources (equal); writing – review and editing (equal). **Preben Clausen:** Conceptualization (equal); resources (equal); writing – review and editing (supporting). **Kathryn M. Dickson:** Conceptualization (equal); resources (equal); writing – review and editing (supporting). **Barwolt S. Ebbinge:** Conceptualization (equal); resources (equal); writing – review and editing (supporting). **Gudmundur A. Gudmundsson:** Conceptualization (equal); resources (equal); writing – review and editing (supporting). **George K. Sage:** Data curation (supporting); investigation (equal); writing – review and editing (supporting). **Jolene R. Rearick:** Investigation (supporting); writing – review and editing (supporting). **Dirk V. Derksen:** Conceptualization (equal); data curation (supporting); funding acquisition (equal); resources (equal); writing – review and editing (equal). **Sandra L. Talbot:** Conceptualization (equal); data curation (equal); formal analysis (supporting); investigation (equal); resources (equal); writing – original draft (supporting).

## FUNDING INFORMATION

This research was funded by the Environment and Climate Change Canada, US Geological Survey, and Washington Brant Foundation.

## CONFLICT OF INTEREST STATEMENT

The authors declare no conflicts of interest.

## Supporting information


Figures S1–S2

Tables S1–S4

## Data Availability

Microsatellite genotype data and sample information are available at Sonsthagen et al. (2024). Mitochondrial control region sequences are available on GenBank (Accession Numbers: PP408688–PP408829).

## References

[ece311245-bib-0001] Abbott, R. J. , Smith, L. C. , Milne, R. I. , Crawford, R. M. M. , Wolff, K. , & Balfour, J. (2000). Molecular analysis of plant migration and refugia in the arctic. Science, 289, 1343–1346. 10958779 10.1126/science.289.5483.1343

[ece311245-bib-0002] Alisauskas, R. T. , Calvert, A. M. , Leafloor, J. O. , Rockwell, R. F. , Drake, K. L. , Kellett, D. K. , Brook, R. W. , & Abraham, K. F. (2022). Subpopulation contributions to a breeding metapopulation of migratory arctic herbivores: Survival, fecundity and asymmetric dispersal. Ecography, 7, e05653.

[ece311245-bib-0003] Baldassare, G. (2014). Ducks, geese, and swans of North America. John Hopkins Press.

[ece311245-bib-0004] Bandelt, H.‐J. , Forster, P. , & Röhl, A. (1999). Median‐joining networks for inferring intraspecific phylogenies. Molecular Biology and Evolution, 16, 37–48. 10331250 10.1093/oxfordjournals.molbev.a026036

[ece311245-bib-0005] Barry, T. W. (1962). Effect of late seasons on Atlantic Brant reproduction. The Journal of Wildlife Management, 26, 19–26.

[ece311245-bib-0006] Beerli, P. , & Felsenstein, J. (1999). Maximum likelihood estimation of migration rates and population numbers of two populations using a coalescent approach. Genetics, 152, 763–773. 10353916 10.1093/genetics/152.2.763 PMC1460627

[ece311245-bib-0007] Beerli, P. , & Felsenstein, J. (2001). Maximum likelihood estimation of a migration matrix and effective population sizes in n subpopulations by using a coalescent approach. Proceedings of the National Academy of Sciences of the United States of America, 98, 4563–4568. 11287657 10.1073/pnas.081068098 PMC31874

[ece311245-bib-0008] Benjamini, Y. , & Yekutieli, D. (2001). The control of false discovery rate in multiple testing under dependency. Annals of Statistics, 29, 1165–1188.

[ece311245-bib-0009] Bertl, J. , Ringbauer, H. , & Blum, M. G. B. (2018). Can secondary contact following range expansion be distinguished from barriers to gene flow? PeerJ, 6, e5325. 30294507 10.7717/peerj.5325 PMC6171497

[ece311245-bib-0010] Bollinger, K. S. , & Derksen, D. V. (1996). Demographic characteristics of molting black brant near Teshekpuk Lake, Alaska. Journal of Field Ornithology, 67, 141–158.

[ece311245-bib-0011] Boyd, H. , & Maltby, L. S. (1979). The brant of the western Queen Elizabeth Islands, N.W.T. In R. L. Jarvis & J. C. Bartonek (Eds.), Management and biology of Pacific flyway geese. Oregon State University Book Stores, Inc.

[ece311245-bib-0012] Boyd, H. , Maltby, L. S. , & Reed, A. (1988). Differences in the plumage patterns of brant breeding in high arctic Canada. Canadian Wildlife Service Progress Note, 174, 1–9.

[ece311245-bib-0013] Boyd, W. S. , Ward, D. H. , Kraege, D. K. , & Gerick, A. A. (2013). Migration patterns of Western High Arctic (Grey‐belly) Brant *Branta bernicla* . Wildfowl Special Issue, 3, 3–25.

[ece311245-bib-0014] Brown, J. I. , Lavretsky, P. , Wilson, R. E. , Haughey, C. L. , Boyd, W. S. , Esler, D. , Talbot, S. L. , & Sonsthagen, S. A. (2020). High fidelity does not equate to population structure and low inter‐specific gene flow for common goldeneye and barrow's goldeneye in North America. Journal of Avian Biology, 51, e02600.

[ece311245-bib-0015] Buchholz, W. , Pearce, J. M. , Pierson, B. J. , & Scribner, K. (1998). Dinucleotide repeat polymorphisms in waterfowl (family Anatidae): Characterization of a sex‐linked (z‐specific) and 14 biparentally inherited loci. Animal Genetics, 29, 323–325. 9745676

[ece311245-bib-0016] Busch, J. D. , Waser, P. M. , & DeWoody, J. A. (2008). Recent demographic bottlenecks are not accompanied by a genetic signature in banner‐tailed kangaroo rats (*Dipodomys spectabilis*). Molecular Ecology, 16, 2450–2462. 10.1111/j.1365-294X.2007.03283.x 17561905

[ece311245-bib-0017] Castelli, P. , Costanzo, G. , Crenshaw, B. , Davies, C. , DiBona, M. , Dickson, K. , Fuller, J. , Hindman, L. , Huang, M. , Lefebvre, J. , Nichols, T. , Osenkowski, J. , Poussart, C. , & Reed, E. (2011). Atlantic brant management plan (p. 34). Atlantic Flyway Council, US Fish and Wildlife Service.

[ece311245-bib-0018] Castelli, P. M. , Dickson, K. M. , & Cramer, D. M. (2010). Spatial and temporal distribution of Atlantic brant . Report to the Atlantic Flyway Council, Atlantic Flyway Office Division of Migratory Bird Management, US Fish and Wildlife Service.

[ece311245-bib-0019] Cathey, J. C. , DeWoody, J. A. , & Smith, L. M. (1998). Microsatellite markers in Canada geese (*Branta canadensis*). Journal of Heredity, 89, 173–175.

[ece311245-bib-0020] Clausen, P. , Madsen, J. , Percival, S. M. , Anderson, G. Q. A. , Koffijberg, K. , Mehlum, F. , & Vangeluwe, D. (1999). Light‐bellied Brent Goose, *Branta bernicla hrota*: Svalbard. In J. Madsen , G. Cracknell , & A. D. Fox (Eds.), Goose populations of the Western Palearctic: A review of status and distribution (pp. 312–327). Wetlands International Publication No. 48, Wetlands International and National Environmental Research Institute.

[ece311245-bib-0021] Cooch, E. , Rockwell, R. F. , & Brault, S. (2001). Retrospective analysis of demographic responses to environmental change: A lesser snow goose example. Ecological Monographs, 71, 377–400.

[ece311245-bib-0022] Corbin, K. W. (1987). In F. Cooke & P. A. Buckley (Eds.), Geographic variation and speciation. Avian genetics: A population and ecological approach. Academic Press Inc.

[ece311245-bib-0023] Cornuet, J.‐M. , & Luikart, G. (1996). Description and power analysis of two tests for detecting recent population bottlenecks from allele frequency data. Genetics, 144, 2001–2014. 8978083 10.1093/genetics/144.4.2001 PMC1207747

[ece311245-bib-0024] Cottam, C. , Lynch, J. J. , & Nelson, A. J. (1944). Food habits and management of American sea brant. Journal of Wildlife Management, 8, 36–56.

[ece311245-bib-0025] Cotter, R. C. , & Hines, J. E. (2001). Breeding biology of brant on Banks Island, Northwest Territories, Canada. Arctic, 54, 357–366.

[ece311245-bib-0026] Cumer, T. , Machado, A. P. , Dumont, G. , Bontzorlos, V. , Ceccherello, R. , Charter, M. , Dichmann, K. , Kassinis, N. , Lourenco, R. , Manzia, F. , Martens, H.‐D. , Prevost, L. , Rakovic, M. , Roque, I. , Siverio, F. , Roulin, A. , & Goudet, J. (2022). Landscape and climatic variations shaped secondary contacts amid Barn Owls of the Western Palearctic. Molecular Biology and Evolution, 39, msab343. 34893883 10.1093/molbev/msab343 PMC8789042

[ece311245-bib-0027] Dalloyau, S. (2022). Brant and barnacle geese wintering in France: Review of the 2021–2022 season. Réseau National Geese.

[ece311245-bib-0028] Di Rienzo, A. , Peterson, A. C. , Garza, J. C. , Valdes, A. M. , Slatkin, M. , & Freimer, N. B. (1994). Mutational processes of simple‐sequence repeat loci in human populations. Proceedings of the National Academy of Sciences of the United States of America, 91, 3166–3170. 8159720 10.1073/pnas.91.8.3166 PMC43536

[ece311245-bib-0029] Dray, S. , & Dufour, A.‐B. (2007). The ade4 package: Implementing the duality diagram for ecologists. Journal of Statistical Software, 22, 1–20.

[ece311245-bib-0030] Earl, D. A. , & vonHoldt, B. M. (2012). STRUCTURE HARVESTER: A website and program for visualizing STRUCTURE output and implementing the Evanno method. Conservation Genetics Resources, 4, 359–361.

[ece311245-bib-0031] Ebbinge, B. S. , Berrevoets, C. , Clausen, P. , Ganter, B. , Günther, K. , Koffijberg, K. , Mahéo, R. , Rowcliffe, M. , Joseph, A. S. , Südbeck, P. , & Syroechkovsky, E. E., Jr. (1999). Dark‐bellied Brent Goose, *Branta bernicla bernicla* . In J. Madsen , G. Cracknell , & A. D. Fox (Eds.), Goose populations of the Western Palearctic: A review of status and distribution. Wetlands International Publication No. 48, Wetlands International and National Environmental Research Institute.

[ece311245-bib-0032] Ebbinge, B. S. , & Spaans, B. (1995). The importance of body reserves accumulated in spring staging areas in the temperate zone for breeding in dark‐bellied brent geese *Branta bernicla bernicla* in the high Arctic. Journal of Avian Biology, 26, 105–113.

[ece311245-bib-0033] Epps, C. W. , & Keyghobadi, N. (2015). Landscape genetics in a changing world: Disentangling historical and contemporary influences and inferring change. Molecular Ecology, 24, 6021–6040. 26547281 10.1111/mec.13454

[ece311245-bib-0034] Erskine, A. J. (1988). The changing patterns of brant migration in eastern North America. Journal of Field Ornithology, 59, 110–119.

[ece311245-bib-0035] Evanno, G. , Regnaut, S. , & Goudet, J. (2005). Detecting the number of clusters of individuals using the software STRUCTURE: A simulation study. Molecular Ecology, 14, 2611–2620. 15969739 10.1111/j.1365-294X.2005.02553.x

[ece311245-bib-0036] Excoffier, L. , Laval, G. , & Schneider, S. (2005). Arlequin ver. 3.0: An integrated software package for population genetics data analysis. Evolutionary Bioinformatics Online, 1, 47–50. PMC2658868 19325852

[ece311245-bib-0037] Fedorov, V. B. , Goropashnaya, A. V. , Jaarola, M. , & Cook, J. A. (2003). Phylogeography of lemmings (*Lemmus*): No evidence for postglacial colonization of Arctic from the Beringian refugium. Molecular Ecology, 12, 725–731. 12675827 10.1046/j.1365-294x.2003.01776.x

[ece311245-bib-0038] Fedorov, V. B. , Goropashnaya, A. V. , Jarrell, G. H. , & Fredga, K. (1999). Phylogeographic structure and mitochondrial DNA variation in true lemmings (*Lemmus*) from the Eurasian Arctic. Biological Journal of the Linnean Society, 66, 357–371.

[ece311245-bib-0039] Fedorov, V. B. , & Stenseth, N. C. (2002). Multiple glacial refugia in the North American arctic: Inference from phylogeography of the collared lemming (*Dicrostonyx groenlandicus*). Proceedings of the Royal Society B: Biological Sciences, 269, 2071–2077. 10.1098/rspb.2002.2126 PMC1691144 12396480

[ece311245-bib-0040] Forster, P. , Torroni, A. , Renfrew, C. , & Röhl, A. (2001). Phylogenetic star contraction applied to Asian and Papuan mtDNA evolution. Molecular Biology and Evolution, 18, 1864–1881. 11557793 10.1093/oxfordjournals.molbev.a003728

[ece311245-bib-0041] Fox, A. D. , & Leafloor, J. O. (Eds.). (2018). A global audit of the status and trends of Arctic and Northern Hemisphere goose populations. Conservation of Arctic Flora and Fauna International Secretariat.

[ece311245-bib-0042] Fu, Y. X. (1997). Statistical tests on neutrality of mutations against population growth, hitchhiking and background selection. Genetics, 147, 915–925. 9335623 10.1093/genetics/147.2.915 PMC1208208

[ece311245-bib-0043] Ganter, B. (2000). Seagrass (*Zostera* spp.) as food for brent geese (*Branta bernicla*): An overview. Helgoland Marine Research, 54, 63–70.

[ece311245-bib-0044] Garza, J. C. , & Williamson, E. G. (2001). Detection of reduction in population size using data from microsatellite loci. Molecular Ecology, 10, 305–318. 11298947 10.1046/j.1365-294x.2001.01190.x

[ece311245-bib-0045] Goudet, J. (1995). FSTAT (ver 1.2): a computer program to calculate F‐statistics. Journal of Heredity, 86, 485–486.

[ece311245-bib-0046] Goudet, J. (2001). *FSTAT, a program to estimate and test gene diversities and fixation indices* (version 2.9.3). https://www2.unil.ch/popgen/softwares/fstat.htm

[ece311245-bib-0047] Griffiths, R. , Double, M. C. , Orr, K. , & Dawson, R. J. G. (1998). A DNA test to sex most birds. Molecular Ecology, 7, 1071–1075. 9711866 10.1046/j.1365-294x.1998.00389.x

[ece311245-bib-0048] Handel, C. M. , Pajot, L. M. , Talbot, S. L. , & Sage, G. K. (2006). Use of buccal swabs for sampling DNA from nestling and adult birds. Wildlife Society Bulletin, 34, 1094–1100.

[ece311245-bib-0049] Hellquist, A. , Waern, M. , & Gerdin, M. (2018). Assortative mating of dark‐bellied brent goose and black brant at Olen yok delta, Russia, in July 2016. Dutch Birding, 40, 318–323.

[ece311245-bib-0050] Hewitt, G. M. (2001). Speciation, hybrid zones and phylogeography—Or seeing genes in space and time. Molecular Ecology, 10, 537–549. 11298967 10.1046/j.1365-294x.2001.01202.x

[ece311245-bib-0051] Hewitt, G. M. (2004). Genetic consequences of climatic oscillations in the Quaternary. Philosophical Transactions of the Royal Society B, 359, 183–195. 10.1098/rstb.2003.1388 PMC1693318 15101575

[ece311245-bib-0052] Jakobsson, M. , & Rosenberg, N. A. (2007). CLUMPP: A cluster matching and permutation program for dealing with label switching and multimodality in analysis of population structure. Bioinformatics, 23, 1801–1806. 17485429 10.1093/bioinformatics/btm233

[ece311245-bib-0053] Jombart, T. (2008). adegenet: A R package for the multivariate analysis of genetic markers. Bioinformatics, 24, 1403–1405. 18397895 10.1093/bioinformatics/btn129

[ece311245-bib-0054] Jones, O. R. , & Wang, J. (2010). COLONY: A program for parentage and sibship inference from multilocus genotype data. Molecular Ecology Resources, 10, 551–555. 21565056 10.1111/j.1755-0998.2009.02787.x

[ece311245-bib-0055] Koffijberg, K. , van Winden, E. , & Clausen, P. (2013). The Netherlands as a winter refuge for light‐bellied brent geese *Branta bernicla hrota* . Wildfowl Special Issue, 3, 40–46.

[ece311245-bib-0056] Kölzsch, A. , Müskens, G. J. D. M. , Szinai, P. , Moonen, S. , Glazov, P. , Kruckenberg, H. , Wikelski, M. , & Nolet, B. A. (2019). Flyway connectivity and exchange primarily driven by moult migration in geese. Movement Ecology, 7, 3. 30733867 10.1186/s40462-019-0148-6 PMC6354378

[ece311245-bib-0057] Lanctot, R. , Goatcher, B. , Scribner, K. , Talbot, S. , Pierson, B. , Esler, D. , & Zwiefelhofer, D. (1999). Harlequin duck recovery from the Exxon Valdez oil spill: A population genetics perspective. The Auk, 116, 781–791.

[ece311245-bib-0058] Leach, A. G. , Ward, D. H. , Sedinger, J. S. , Riecke, T. V. , Hupp, J. W. , & Ritchie, R. J. (2018). Spatial distribution of band recoveries of black brant. The Journal of Wildlife Management, 83, 304–311.

[ece311245-bib-0059] Lewis, T. L. , Ward, D. H. , Sedinger, J. S. , Reed, A. , Derksen, D. V. , Carboneras, C. , Christie, D. A. , & Kirwan, G. M. (2020). Brant (*Branta bernicla*), version 1.0. In S. M. Billerman (Ed.), Birds of the world. Cornell Lab of Ornithology.

[ece311245-bib-0060] Lindberg, M. S. , Sedinger, J. S. , Derksen, D. V. , & Rockwell, R. F. (1998). Natal and breeding philopatry in a black brant, *Branta bernicla nigricans*, metapopulation. Ecology, 79, 1893–1904.

[ece311245-bib-0061] Luque, G. M. , Vayssade, C. , Facon, B. , Guillemaud, T. , Courchamp, F. , & Fauvergue, X. (2016). The genetic allee effect: A unified framework for the genetics and demography of small populations. Ecosphere, 7, e01413.

[ece311245-bib-0062] Maak, S. , Neumann, K. , von Lengerken, G. , & Gattermann, R. (2000). First seven microsatellites developed for the Peking duck (*Anas platyrhynchos*). Animal Genetics, 31, 233. 10895321

[ece311245-bib-0063] Maak, S. , Wimmer, K. , Weigend, S. , & Neumann, K. (2003). Isolation and characterization of 18 microsatellites in the Peking duck (*Anas platyrhynchos*) and their application in other waterfowl species. Molecular Ecology Notes, 3, 224–227.

[ece311245-bib-0064] Macdonald, C. A. , Fraser, K. C. , Gilchrist, H. G. , Kyser, T. K. , Fox, J. W. , & Love, O. P. (2012). Strong migratory connectivity in a declining Arctic passerine. Animal Migration, 1, 23–30.

[ece311245-bib-0065] MacPherson, A. H. (1965). The origin of diversity in mammals of the Canadian arctic tundra. Systematic Zoology, 14, 153–173.

[ece311245-bib-0066] Maier, P. A. , Vandergast, A. G. , Ostoja, S. M. , Aguilar, A. , & Bohonak, A. J. (2019). Pleistocene glacial cycles drove lineage diversification and fusion in the Yosemite toad (*Anaxyrus canorus*). Evolution, 73, 2476–2496. 31661155 10.1111/evo.13868

[ece311245-bib-0067] Maruyama, T. , & Fuerst, P. A. (1985). Population bottlenecks and nonequilibrium models in population genetics. II. Number of alleles in a small population that was formed by a recent bottleneck. Genetics, 111, 675–689. 4054612 10.1093/genetics/111.3.675 PMC1202664

[ece311245-bib-0068] Narum, S. R. (2006). Beyond Bonferroni: Less conservative analyses for conservation genetics. Conservation Genetics, 7, 783–787.

[ece311245-bib-0069] O'Briain, M. , Reed, A. , & Macdonald, S. D. (1998). Breeding, moulting, and site fidelity of brant (*Branta bernicla*) on Bathurst and Seymour islands in the Canadian high arctic. Arctic, 51, 350–360.

[ece311245-bib-0070] Oetting, W. S. , Lee, H. K. , Flanders, D. J. , Weisner, G. L. , Sellers, T. A. , & King, R. A. (1995). Linkage analysis with multiplexed short tandem repeat polymorphisms using infrared fluorescence and M13 tailed primers. Genomics, 30, 450–458. 8825630 10.1006/geno.1995.1264

[ece311245-bib-0071] Ohta, T. , & Kimura, M. (1973). A model of mutation appropriate to estimate the number of electrophoretically detectable alleles in a finite population. Genetical Research, 22, 201–204. 4777279 10.1017/s0016672300012994

[ece311245-bib-0072] Olson, S. M. C. (2021). Pacific flyway data book, 2021. U.S. Department of Interior, Fish and Wildlife Service, Division of Migratory Bird Management.

[ece311245-bib-0073] Ottenburghs, J. , Megens, H.‐J. , Kraus, R. H. , Madsen, O. , van Hooft, P. , van Wieren, S. E. , Crooijmans, R. P. , Ydenberg, R. C. , Groenen, M. A. , & Prins, H. H. (2016). A tree of geese: A phylogenomic perspective on the evolutionary history of true geese. Molecular Phylogenetics and Evolution, 101, 303–313. 27233434 10.1016/j.ympev.2016.05.021

[ece311245-bib-0074] Pacific Flyway Council . (2018). Management plan for the Pacific population of brant (p. 48). Pacific Flyway Council, Care of U.S. Fish and Wildlife Service, Division of Migratory Bird Management.

[ece311245-bib-0075] Paulus, K. B. , & Tiedemann, R. (2003). Ten polymorphic autosomal microsatellite loci for the eider duck *Somateria mollissima* and their cross‐species applicability among waterfowl species (Anatidae). Molecular Ecology Notes, 3, 250–252.

[ece311245-bib-0076] Pennycuick, C. J. , Griffin, L. R. , Colhoun, K. , & Angwin, R. (2011). A trial of a non‐statistical computer program for monitoring fuel reserves, response to wind and other details from GPS tracks of migrating geese. Journal of Ornithology, 152, 87–99.

[ece311245-bib-0077] Pielou, E. C. (1991). After the ice age: The return of life to glaciated North America. University of Chicago Press.

[ece311245-bib-0078] Piry, S. , Luikart, G. , & Cornuet, J. M. (1999). BOTTLENECK: A computer program for detecting recent reductions in the effective population size using allele frequency data. Journal of Heredity, 90, 502–503.

[ece311245-bib-0079] Ploeger, P. L. (1968). Geographical differentiation in arctic Anatidae as a result of isolation during the last glacial period. Ardea, 56, 1–159.

[ece311245-bib-0080] Portenko, L. A. (1981). Birds of the Chukchi Peninsula and Wrangel Island (Vol. 1). Amerind Publishing Co, PVT. (translated from Russian).

[ece311245-bib-0081] Pritchard, J. , Stephens, M. , & Donnelly, P. (2000). Inference of population structure using multilocus genotype data. Genetics, 155, 945–959. 10835412 10.1093/genetics/155.2.945 PMC1461096

[ece311245-bib-0082] R Core Team . (2020). R: A language and environment for statistical computing. R Foundation for Statistical Computing. https://www.R‐project.org/

[ece311245-bib-0083] Raymond, M. , & Rousset, F. (1995). GENEPOP (Version 1.2): Population genetics software for exact tests and ecumenicism. Journal of Heredity, 86, 248–249.

[ece311245-bib-0084] Reed, A. , Davison, M. A. , & Kraege, D. K. (1989). Segregation of brent geese *Branta bernicla* wintering and staging in Puget Sound and the Strait of Georgia. Wildfowl, 40, 22–31.

[ece311245-bib-0085] Reed, A. , Stehn, R. , & Ward, D. (1989). Autumn use of Izembek Lagoon, Alaska, by brant from different breeding areas. *The* Journal of Wildlife Management, 53, 720–725.

[ece311245-bib-0086] Roberts, A. J. , Dooley, J. L. , Ross, B. E. , Nichols, T. C. , Leafloor, J. O. , & Dufour, K. W. (2021). An integrated population model for harvest management of Atlantic brant. The Journal of Wildlife Management, 85, 897–908.

[ece311245-bib-0087] Rogers, J. P. (1979). *Branta bernicla hrota* in the USA—A management review. In M. Smart (Ed.), Proceedings of the First Technical Meeting on Western Palearctic Migratory Bird Management, Paris, France. International Waterfowl Research Bureau.

[ece311245-bib-0088] Rousset, F. (2008). GENEPOP'007: A complete reimplementation of the GENEPOP software for Windows and Linux. Molecular Ecology Resources, 8, 103–106. 21585727 10.1111/j.1471-8286.2007.01931.x

[ece311245-bib-0089] Ruokonen, M. , Kvist, L. , & Lumme, J. (2000). Close relatedness between mitochondrial DNA from seven *Anser* goose species. Journal of Evolutionary Biology, 13, 532–540.

[ece311245-bib-0090] Sawa, Y. , Sato, T. , Ikeuchi, T. , & Pozdnyakov, V. (2019). Banding survey at colonies of brent goose, *Branta bernicla* in the Lena Delta, Russia, and a recovery record. The Bulletin of the Japanese Bird Banding Association, 31, 53–64.

[ece311245-bib-0091] Sawa, Y. , Tamura, C. , Ikeuchi, T. , Fujii, K. , Ishioroshi, A. , Shimada, T. , Tatsuzawa, S. , Deng, X. , Cao, L. , Kim, H. , & Ward, D. (2020). Migration routes and population status of the brent goose *Branta bernicla nigricans* wintering in East Asia. Wildfowl, 6, 244–266.

[ece311245-bib-0092] Scribner, K. T. , Talbot, S. L. , Pearce, J. M. , Pierson, B. J. , Bollinger, K. S. , & Derksen, D. V. (2003). Phylogeography of Canada geese (*Branta canadensis*) in western North America. The Auk, 140, 889–907.

[ece311245-bib-0093] Sedinger, J. S. , Riecke, T. V. , Leach, A. G. , & Ward, D. H. (2019). The black brant population is declining based on mark recapture. The Journal of Wildlife Management, 83, 627–637.

[ece311245-bib-0094] Sedinger, J. S. , Ward, D. H. , Anthony, R. M. , Derksen, D. V. , Lensink, C. J. , & Bollinger, K. S. (1994). Management of the Pacific black brant: Population structure and conservation issues. Transactions of the North American Wildlife and Natural Resources Conference, 59, 50–62.

[ece311245-bib-0095] Sedinger, J. S. , Ward, D. H. , Schamber, J. L. , Butler, W. I. , Eldridge, W. D. , Conant, B. , Voelzer, J. F. , Chelgren, N. D. , & Herzog, M. P. (2006). Effects of El Niño on distribution and reproductive performance of Black Brant. Ecology, 87, 151–159. 16634306 10.1890/04-1013

[ece311245-bib-0096] Shaw, A. K. (2020). Causes and consequences of individual variation in animal movement. Movement Ecology, 8, 12. 32099656 10.1186/s40462-020-0197-x PMC7027015

[ece311245-bib-0097] Shields, G. F. (1990). Analysis of mitochondrial DNA of Pacific black brant (*Branta bernicla nigricans*). The Auk, 107, 620–623.

[ece311245-bib-0098] Shorey, R. I. (2005). *Phylogeographies of snow, Ross's, Canada, and cackling geese: Defining spatial structure of biological diversity* (PhD). Michigan State University.

[ece311245-bib-0099] Snell‐Rood, E. C. , & Steck, M. K. (2019). Behaviour shapes environmental variation and selection on learning and plasticity: Review of mechanisms and implications. Animal Behaviour, 147, 147–156.

[ece311245-bib-0100] Sonsthagen, S. A. , Talbot, S. L. , Scribner, K. , & McCracken, K. (2011). Multilocus phylogeography and population structure of common eiders breeding in North America and Scandinavia. Journal of Biogeography, 38, 1368–1380.

[ece311245-bib-0101] Sonsthagen, S. A. , Wilson, R. E. , Lavretsky, P. , & Talbot, S. L. (2019). Coast to coast: High genomic connectivity in North American scoters. Ecology and Evolution, 9, 7246–7261. 31380047 10.1002/ece3.5297 PMC6662410

[ece311245-bib-0102] Sonsthagen, S. A. , Wilson, R. E. , Pierson, B. J. , Sage, G. K. , Talbot, S. L. , Derksen, D. V. , & Ward, D. H. (2024). Branta (Branta bernicla) genetic data from North America, Europe, and Asia: U.S. Geological Survey data release . 10.5066/P96G9LAJ

[ece311245-bib-0103] Sorenson, M. D. , & Fleischer, R. C. (1996). Multiple independent transpositions of mitochondrial DNA control region sequences to the nucleus. Proceedings of the National Academy of Sciences of the United States of America, 93, 15239–15245. 8986794 10.1073/pnas.93.26.15239 PMC26387

[ece311245-bib-0104] Sorenson, M. D. , & Quinn, T. W. (1998). Numts: A challenge for avian systematics and population biology. The Auk, 115, 214–221.

[ece311245-bib-0105] Stewart, J. R. , Lister, A. M. , Barnes, I. , & Dalén, L. (2010). Refugia revisited: Individualistic responses of species in space and time. Proceedings of the Royal Society B: Biological Sciences, 277, 661–671. 10.1098/rspb.2009.1272 PMC2842738 19864280

[ece311245-bib-0106] Syroechkovski, E. E. , Zöckler, C. , & Lappo, E. (1998). Status of brent goose in northwest Yakutia, east Siberia. British Birds, 91, 565–571.

[ece311245-bib-0107] Taberlet, P. , Fumagalli, L. , Wust‐Saucy, A.‐G. , & Cosson, J.‐F. (1998). Comparative phylogeography and postglacial colonization routes in Europe. Molecular Ecology, 7, 453–464. 9628000 10.1046/j.1365-294x.1998.00289.x

[ece311245-bib-0108] Tajima, F. (1989). The amount of DNA polymorphism maintained in a finite population when the neutral mutation rate varies among sites. Genetics, 143, 1457–1465. 10.1093/genetics/143.3.1457 PMC1207412 8807315

[ece311245-bib-0109] Talbot, S. L. , Palmer, A. G. , Sage, G. K. , Sonsthagen, S. A. , Swem, T. , Brimm, D. J. , & White, C. M. (2011). Lack of genetic polymorphism among peregrine falcons *Falco peregrinus* of Fiji. Journal of Avian Biology, 42, 414–428.

[ece311245-bib-0110] Tremblay, N. O. , & Schoen, D. J. (1999). Molecular phylogeography of *Dryas integrifolia*: Glacial refugia and postglacial recolonization. Molecular Ecology, 8, 1187–1198. 10447859 10.1046/j.1365-294x.1999.00680.x

[ece311245-bib-0111] Wang, J. (2017). The computer program structure for assigning individuals to populations: Easy to use but easier to misuse. Molecular Ecology Resources, 17, 981–990. 28028941 10.1111/1755-0998.12650

[ece311245-bib-0112] Ward, D. H. , Amundson, C. L. , Stehn, R. A. , & Dau, C. P. (2018). Long‐term trends in fall age ratios of black brant. The Journal of Wildlife Management, 82, 362–373.

[ece311245-bib-0113] Ward, D. H. , Derksen, D. V. , Kharitonov, S. P. , Stishov, M. , & Baranyuk, V. V. (1993). Status of Pacific Black Brant *Branta bernicla nigricans* on Wrangel Island, Russian Federation. Wildfowl, 44, 39–48.

[ece311245-bib-0114] Ward, D. H. , Reed, A. , Sedinger, J. S. , Black, J. M. , Derksen, D. V. , & Castelli, P. M. (2005). North American brant: Effects of changes in habitat and climate on population dynamics. Global Change Biology, 11, 869–880.

[ece311245-bib-0115] Williams, M. , Dunkerley, D. , De Deckker, P. , Kershaw, P. , & Chappel, J. (1998). Quaternary environments. Oxford University Press.

[ece311245-bib-0116] Wilson, R. E. , Ely, C. , & Talbot, S. L. (2018). Flyway structure in the circumpolar greater white‐fronted goose. Ecology and Evolution, 8, 8490–8507. 30250718 10.1002/ece3.4345 PMC6144976

[ece311245-bib-0117] Wilson, R. E. , Gust, J. R. , Petersen, M. R. , & Talbot, S. L. (2016). Spatial genetic structure in long‐tailed ducks (*Clangula hyemalis*) among Russian, Alaskan, and Canadian breeding populations. Arctic, 69, 65–78.

[ece311245-bib-0118] Wilson, R. E. , Sonsthagen, S. A. , DaCosta, J. M. , Sorenson, M. D. , Fox, A. D. , Weaver, M. , Skalos, D. , Kondratyev, A. V. , Scribner, K. T. , Walsh, A. , Ely, C. R. , & Talbot, S. L. (2022). As the goose flies: Migration routes and timing influence patterns of genetic diversity in a circumpolar migratory herbivore. Diversity, 14, 1067.

