# Supplementary material for: Where east meets west: Phylogeography of the high Arctic North American brant goose
Source: Ecol Evol. 2024 Apr 10;14(4):e11245. doi: 10.1002/ece3.11245 (PMC11004662; doi:10.1002/ece3.11245)
Supplement: Supplementary file 1 — Figures S1–S2 [file ECE3-14-e11245-s001.pdf]

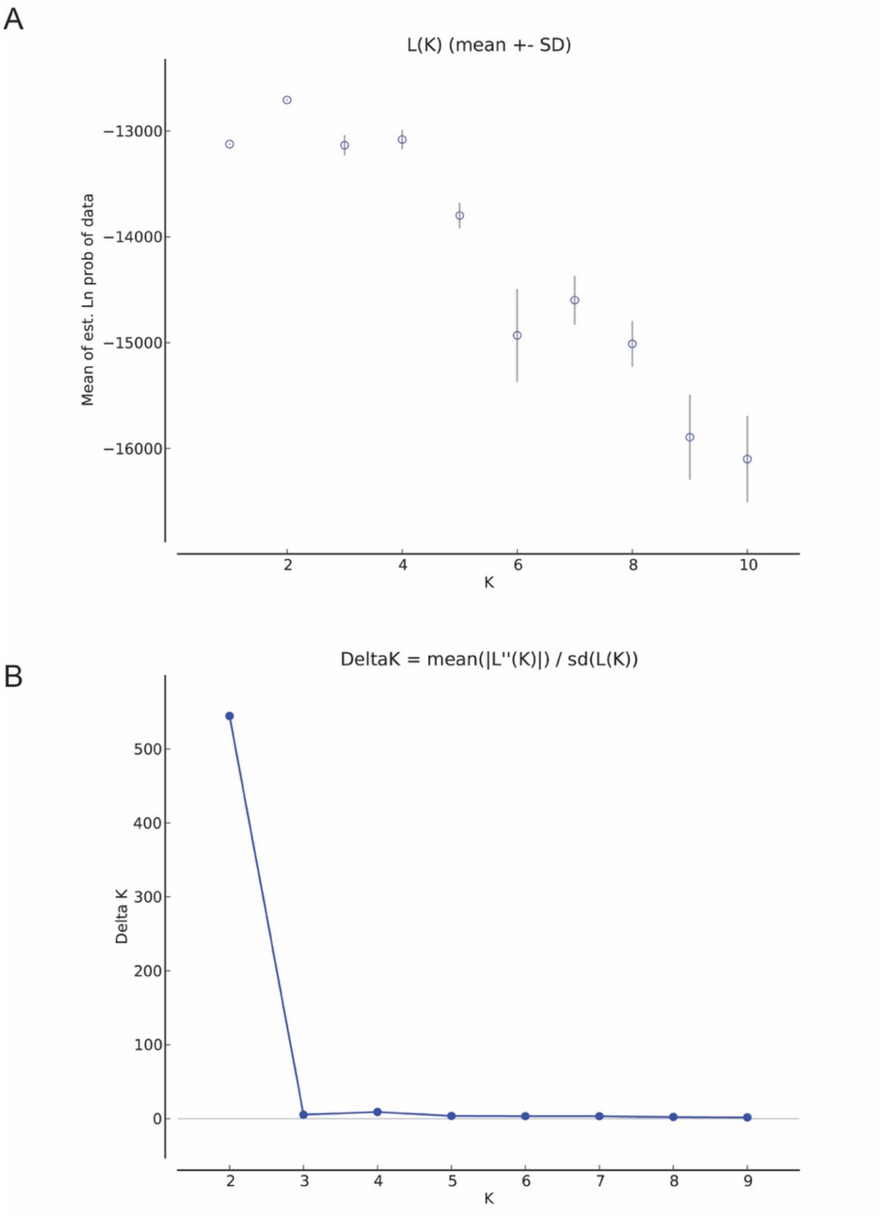

Supplemental Figure 1. STRUCTURE HARVESTER web v.0.6.94 (Earl & vonHoldt, 2012) results showing (A) LN P(K) with standard deviation for each K (number of populations) value and (B) Delta K values following Evanno's method (Evanno 2005). Results are from a STRUCTURE (Pritchard et al., 2000) analysis for brant (*Branta bernicla*) using 12 microsatellites (Sonsthagen et al., 2024). The analysis was repeated 10 times for each K value.

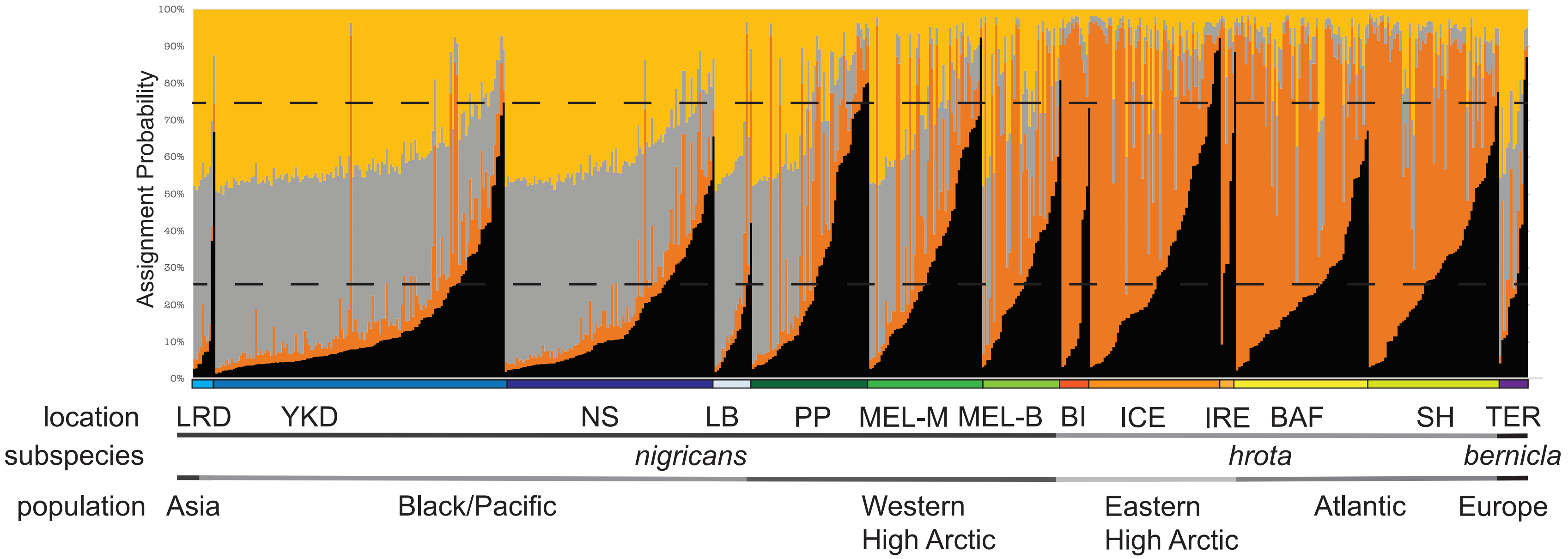

Supplemental Figure 2. STRUCTURE analysis of brant (*Branta bernicla*) showing average assignment probability based on microsatellite data for K = 4. Color blocks under plot correspond to color circles (molting or nesting sample locations) or squares (staging or wintering sample locations) in Figure 2 in main text. Collection location abbreviations are as follows: LRD (Lena River Delta, Russia), YKD (Yukon-Kuskokwim Delta, Alaska), NS (North Slope, Alaska), LB (Liverpool Bay, Canada), PP (Prince Patrick Island, Canada), MEL-M (Melville Island, Canada – Molting), MEL-B (Melville Island, Canada – breeding), BI (Bathurst Island, Canada), ICE (Iceland), IRE (Ireland), BAF (Baffin Island, Canada), SH (Southampton Island, Canada), TER (Terschelling, The Netherlands). Subspecies of *B. bernicla* are indicated as well as designated population or ecological stock.

References

Earl, D. A., & vonHoldt, B. M. (2012). STRUCTURE HARVESTER: a website and program for visualizing STRUCTURE output and implementing the Evanno method. *Conservation Genetics Resources* 4: 359–361.

Evanno, G., Regnaut, S., & Goudet, J. (2005). Detecting the number of clusters of individuals using the software STRUCTURE: A simulation study. *Molecular Ecology* 14: 2611–2620.

Pritchard, J., Stephens, M., & Donnelly, P. (2000). Inference of population structure using multilocus genotype data. *Genetics* 155: 945–959.

Sonsthagen, S. A., Wilson, R. E., Pierson, B. J., Sage, G. K., Talbot, S. L., Derksen, D. V., & Ward, D. H. (2024). Branta (*Branta bernicla*) genetic data from North America, Europe, and Asia: U.S. Geological Survey data release, https://doi.org/10.5066/P96G9LAJ.

Any use of trade, firm, or product names is for descriptive purposes only and does not imply endorsement by the U.S. Government.
